# EGR1 recruits TET1 to shape the brain methylome during development and upon neuronal activity

**DOI:** 10.1038/s41467-019-11905-3

**Published:** 2019-08-29

**Authors:** Zhixiong Sun, Xiguang Xu, Jianlin He, Alexander Murray, Ming-an Sun, Xiaoran Wei, Xia Wang, Emmarose McCoig, Evan Xie, Xi Jiang, Liwu Li, Jinsong Zhu, Jianjun Chen, Alexei Morozov, Alicia M. Pickrell, Michelle H. Theus, Hehuang Xie

**Affiliations:** 1 Fralin Life Sciences Institute at Virginia Tech, Blacksburg, VA 24061 USA; 2 Department of Biological Sciences, Virginia Tech, Blacksburg, VA 24061 USA; 3 Department of Biomedical Sciences and Pathobiology, Virginia-Maryland College of Veterinary Medicine, Blacksburg, VA 24061 USA; 4 School of Neuroscience, Virginia Tech, Blacksburg, VA 24061 USA; 5 Department of Pharmacology, and Bone Marrow Transplantation Center of the First Affiliated Hospital, Zhejiang University School of Medicine, Hangzhou, Zhejiang 310058 China; 6 Institute of Hematology, Zhejiang University & Zhejiang Engineering Laboratory for Stem Cell and Immunotherapy, Hangzhou, Zhejiang 310058 China; 7 Department of Biochemistry, Virginia Tech, Blacksburg, VA 24061 USA; 8 Department of Systems Biology, Beckman Research Institute of City of Hope, Monorovia, CA 91016 USA; 9 Department of Psychiatry and Behavioral Medicine, Virginia Tech Carilion School of Medicine, Roanoke, VA 24016 USA; 10 Virginia Tech Carilion Research Institute, Roanoke, VA 24016 USA; 11 School of Biomedical Engineering and Sciences, Blacksburg, VA 24061 USA

**Keywords:** Neuroscience, Epigenetics in the nervous system, Epigenetics and plasticity

## Abstract

Life experience can leave lasting marks, such as epigenetic changes, in the brain. How life experience is translated into storable epigenetic information remains largely unknown. With unbiased data-driven approaches, we predicted that *Egr1*, a transcription factor important for memory formation, plays an essential role in brain epigenetic programming. We performed EGR1 ChIP-seq and validated thousands of EGR1 binding sites with methylation patterns established during postnatal brain development. More specifically, these EGR1 binding sites become hypomethylated in mature neurons but remain heavily methylated in glia. We further demonstrated that EGR1 recruits a DNA demethylase TET1 to remove the methylation marks and activate downstream genes. The frontal cortices from the knockout mice lacking *Egr1* or *Tet1* share strikingly similar profiles in both gene expression and DNA methylation. In summary, our study reveals EGR1 programs the brain methylome together with TET1 providing new insight into how life experience may shape the brain methylome.

## Abstract (web summary)

It is unclear why neuronal activity induced methylation changes are limited to specific loci in the genome. Here, authors show that the DNA demethylation enzyme, TET1, gains its specificity via the interaction with EGR1, a sequence specific DNA binding protein.

## Introduction

It has been well acknowledged that early postnatal experience is critical for brain development and may induce long-lasting epigenetic changes in postmitotic neurons^1,2^. Growing evidence indicates that learning and memory are highly dependent on the function of epigenetic machinery such as DNA methyltransferases (DNMTs)^3–5^ and DNA demethylases^6–9^, the Ten-Eleven Translocation (Tet) proteins including TET1, TET2, and TET3. Double knockout of *Dnmt1 *and *Dnmt3a* leads to abnormal gene expression contributing to synaptic plasticity and learning and memory deficits^3^. Genetic deletion or knock-down of each TET enzyme results in a unique set of phenotypes^10,11^. *Tet1* is involved in neural progenitor cell proliferation^12^ and neuronal activity-induced active DNA demethylation in the dentate gyrus of the adult mouse brain^13^. *Tet1* knockout mice exhibited impaired hippocampal neurogenesis, significant deficiency in short-term memory retention^12^, abnormal long-term depression and impaired memory extinction^9^. The deletion of *Tet3* leads to neonatal lethality^14^ and neural progenitor cells induced from *Tet3* knockout ES cells undergo apoptosis rapidly with reduced terminal differentiation of neurons^15^. Significant impairment in fear extinction memory was observed in mice with *Tet3* knockdown via shRNA^16^. Although little is known about the role of *Tet2* in neuronal differentiation or function, *Tet2* knockout mice show abnormal hyper-methylation in the frontal cortex^17^. Despite the known needs of DNMTs and TETs for learning and memory, how these enzymes are directed to specific genomic loci in neurons remain elusive.

Neuronal activity-induced DNA methylation changes may occur within hours after electroconvulsive stimulation^18^. This suggests that neurons can react to environmental stimuli and guide the epigenetic machinery to desired genomic loci swiftly. As an immediate early gene, *Egr1* (*Egr1* in mice, *EGR1* in humans, also known as* Zif268*, *Krox-24*, and *NGFI-A*) can be rapidly and transiently induced by neuronal activity^19,20^. *Egr1* is a critical transcriptional regulator involved in brain development, learning, and long-term neuronal plasticity^21–24^. With a rapid increase in expression during the first few weeks after birth, *Egr1* controls the selection, maturation and functional integration of newborn neurons^21^. A seminal study has established a link between maternal care and methylation programming during early postnatal brain development, and *Egr1* was proposed to be an epigenetic regulator of glucocorticoid receptor^1^. More interestingly, EGR1 has a binding motif containing CpG dinucleotides (5′- GCGTGGGCG-3′)^25^ and the binding of EGR1 to target DNA is insensitive to methylation^26,27^. However, whether EGR1 can direct epigenetic machinery to its target sites upon neuronal activation is unknown.

Recently, we have implemented a nonparametric Bayesian clustering approach^28^ to identify genomic loci with bipolar DNA methylation patterns: the presence of both hypo-methylated and hyper-methylated patterns within a mixed cell population. In other words, for sequence reads mapped to a bipolar methylated locus, some of them are completely methylated while others could be completely unmethylated. With this approach, we observed the number of bipolar methylated loci increased dramatically during early stages of brain development and brain bipolar methylated loci were enriched for GWAS variants associated with neurological disorder-related diseases/traits^29^. Interestingly, genes associated with brain bipolar methylated loci are involved in neuronal differentiation, cell migration and cell morphogenesis. In this study, we explored the epigenetic regulatory mechanism underlying the birth of bipolar methylated loci and identified EGR1 as a key mediator involved in brain epigenome programming during postnatal development. Our study provides the first compelling data demonstrating EGR1 recruits TET1 to demethylate EGR1 binding sites. Our results implicate the interaction between transcription factors (TFs) and epigenetic machinery as a general mechanism to achieve locus-specific epigenetic regulation upon neuronal activation.

## Results

### EGR1 peaks lose methylation during brain development

To explore epigenetic regulatory mechanisms during brain development, we followed our previous approach^29^ (see “Methods” for details) to re-analyze methylomes for frontal cortices at different developmental stages and identified a total of 11,178 (human) and 4692 (mouse) bipolar methylated loci within 10 kb upstream and downstream from transcription start sites (TSSs). For these bipolar methylated loci, we determined the methylation correlations between all possible pairs (Supplementary Fig. 1a and 1d) and identified five major co-methylated modules showing distinct methylation profiles during brain development and neural cell specification (Supplementary Fig. 1b and 1e). For instance, in mouse frontal cortices, the bipolar methylated loci in module I and II were hypomethylated in neurons. In contrast, the bipolar methylated loci in module III and IV were found to be hypomethylated in non-neuronal cells, while the bipolar methylated loci in module V tended to show age-related methylation. Using HOMER^30^, we determined the motifs for TFs enriched in each co-methylated module (Supplementary Fig. 1c, 1f and Supplementary Data 1) and identified *Egr1* is associated with module I, the largest module for both human and mouse. More interestingly, the CpG dinucleotides within the EGR1 binding motifs are gradually demethylated during postnatal brain development and the methylation losses are limited in neurons (Supplementary Fig. 2).

**Fig. 1:**
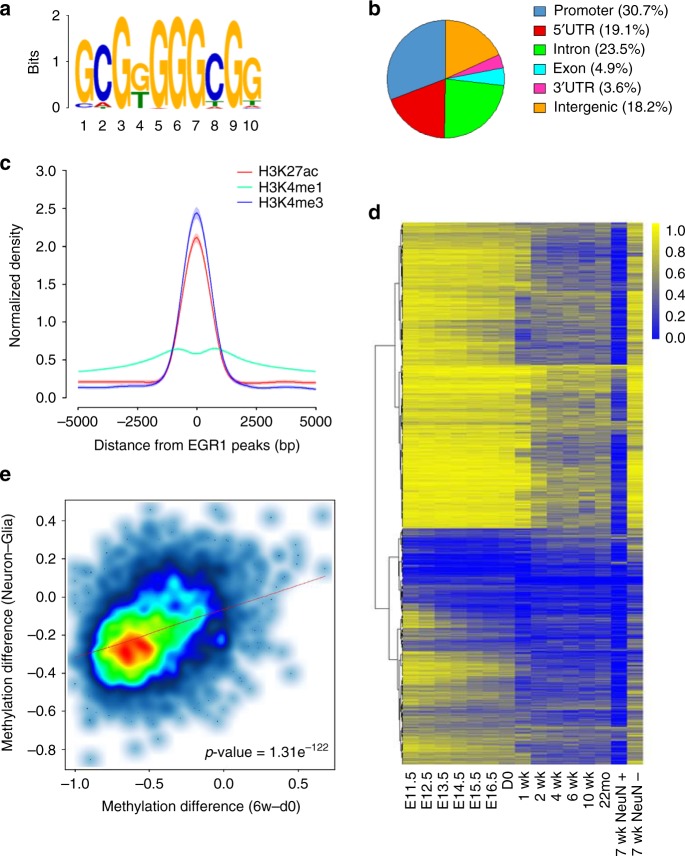
Methylation dynamics of EGR1 binding sites during mouse brain development. **a** EGR1 binding motif (*E* value = 1.1e^−252^) identified from ChIP-seq data generated with mouse adult frontal cortices. **b** Genomic distribution of EGR1 peaks. **c** Distribution of histone marks H3K27ac, H3K4me1, and H3K4me3 surrounding EGR1 peaks. **d** Methylation dynamics of EGR1 binding sites during brain development from embryonic day 11.5 to 22 months, in neurons (NeuN+), and non-neuronal cells (NeuN−). **e** Correlation of methylation changes at EGR1 binding sites during mouse brain development and between cell specification. Only binding sites with at least ten methylation calls in all four methylomes were included. *P*-values were determined with Wilcoxon Rank Sum Test

To validate such a computational prediction, we performed ChIP-seq for EGR1 in duplicate with mouse frontal cortices at 6 weeks and identified 12,014 high-confidence peaks (Supplementary Fig. 3). Independent ChIP-qPCR assays were performed for six loci to confirm the significant enrichment of EGR1 binding in peaks identified (Supplementary Fig. 4a). From the sequences of EGR1 peaks, we determined the most significantly enriched motif as “GCGGGGGCGG” (Fig. 1a, *E*-value = 1.1e^−252^), which is similar to the canonical EGR1 response element reported previously^31^. A total of 81.8% of EGR1 peaks localize in gene promoters (from TSSs to 2 kb upstream of TSSs) or within genic regions (Fig. 1b), and the frequency of EGR1 binding sites increases when approaching transcription start sites (Supplementary Fig. 4b). We further integrated EGR1 peaks with ChIP-seq datasets for histone modifications^32^ and observed that active enhancer mark H3K27ac and active promoter mark H3K4me3 are strongly enriched in the vicinity of EGR1 peaks (Fig. 1c). More specifically, 46.9% and 44.4% of EGR1 binding sites overlap with H3K27ac peaks and H3K4me3 peaks, respectively. Additional ChIP-qPCR assays validated that the H3K27ac mark is enriched at the six genomic loci with EGR1 binding (Supplementary Fig. 4c). These results suggest that EGR1 mainly binds active promoters or enhancers to activate the expression of downstream genes in the mouse frontal cortex.

To examine methylation dynamics of EGR1 peaks, we made use of embryonic forebrain methylomes recently released by ENCODE to obtain the methylation profiles for 12,014 EGR1 peaks during brain development. 51.0% of EGR1 binding sites show constant hypomethylation (methylation level <= 0.2) throughout all developmental stages with methylomes available from E11.5 to 22 m (Supplementary Fig. 5). On the other hand, 34.3% of EGR1 binding sites (*n* = 4,125) exhibit methylation dynamics during development with the maximum methylation difference between stages greater than or equal to 0.2 (The range of methylation was defined from 0 to 1 in this study). The majority of these EGR1 binding sites show decreased methylation changes during development and become hypomethylated in neurons (Fig. 1d). We next focused on the comparisons of DNA methylation between d0 and 6-week-old frontal cortices, and between NeuN+ and NeuN- cells at 7 week. Out of the 4,125 EGR1 binding sites with methylation variation, 2,106 (51.1%) showed methylation decrease by at least 20% during postnatal development (d0 to 6 week) and 3,451 (83.7%) showed methylation decrease by at least 20% in neurons compared with those in glial cells. In contrast, only 111 (2.7%) and 37 (0.8%) of EGR1 binding sites showed methylation increase by at least 20% during development or in neurons, respectively. We next asked if the methylation changes on EGR1 binding sites during development and between the two cell types were correlated. We found that 1,925 out of 2,106 (91.4%) EGR1 binding sites hypomethylated in adult frontal cortex were also hypomethylated in neurons. In addition, the methylation changes in d0 vs. 6 week, and glia vs. neuron were significantly positively correlated (Fig. 1e, Pearson’s *R* = 0.35, *p*-value = 1.31e^−122^). To determine if the 1,925 EGR1 peaks are linked to specific functions, we performed Gene Ontology (GO) enrichment analysis and found that genes with TSS flanking 10 kb of these EGR1 peaks are significantly enriched in the regulation of ion membrane transport, which is important for membrane potential formation and action potential propagation in neurons (Supplementary Fig. 6 and Supplementary Data 2).

### EGR1 interacts and recruits TET1 to its target binding sites

A recent report indicated that a member of EGR family, WT1 (Wilms tumor suppressor gene 1) may recruit TET2 to demethylate its binding sites in leukemia cells^33^. More specifically, the zinc-finger domain (residues 323–449) of WT1 binds directly to the CD domain (C-terminal region) of TET2 enzyme. WT1 and EGR1 share a similar structure and bind to a same consensus DNA sequence^34,35^. Interestingly, the three TET family members also share significant homology^36^. These findings raise the possibility that EGR1 may interact with TET enzymes to program the brain methylome. To test this hypothesis, we reanalyzed RNA-seq data^17^ for mouse frontal cortices to examine the expression profiles of *Egr1* and *Tet* gene family (*Tet1-3*) during mouse brain development. *Egr1* transcript in mouse frontal cortices rapidly increased during fetal to 2-weeks, maintaining at a higher level throughout later developmental stages (Supplementary Fig. 7a). The levels of *Tet2* and *Tet3* expression gradually decrease during development while *Tet1* shows an increased expression level during the second postnatal week (Supplementary Fig. 7b). We further examined the methylation profiles of EGR1 binding sites in *Tet2* knockout mice^17^. It has been reported that 19.7% of regions hypo-methylated in adult frontal cortex vs. fetal are with increased methylation in adult *Tet2* knockout mice^17^. Interestingly, we found that EGR1 binding sites show no significant methylation difference (Wilcoxon rank-sum test) between *Tet2−/−* and wild-type mice (Supplementary Fig. 8). This indicates that *Tet2* is not required for the demethylation of EGR1 binding sites.

**Fig. 2:**
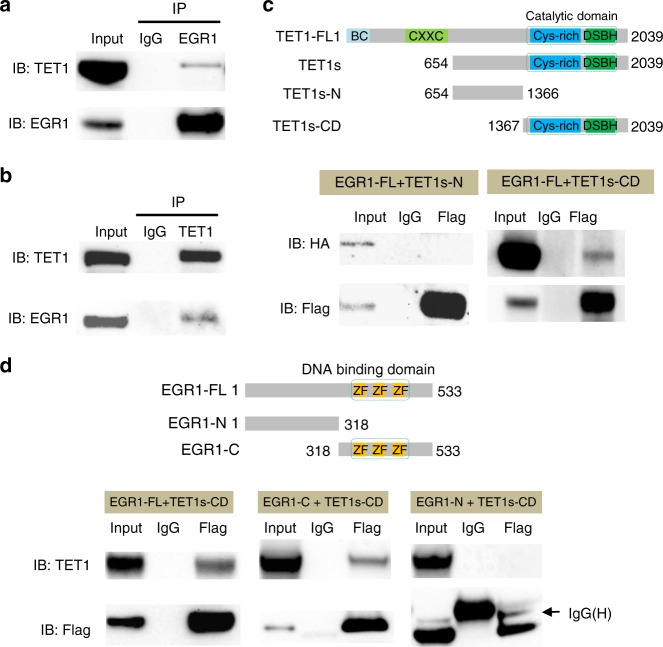
Identification of the protein-protein interaction between EGR1 and TET enzymes by co-immunoprecipitation. **a**, **b** Endogenous association of EGR1 and TET proteins. EGR1 was immuno-precipitated from mouse frontal cortex, followed by western blot to detect TET1 (**a**). TET1 was immuno-precipitated from mouse frontal cortex, followed by western blot to detect EGR1 (**b**). Normal rabbit IgG served as a negative control for immunoprecipitation. IP, immunoprecipitation. **c** Interactions between full-length EGR1 (EGR1-FL) and TET1 deletion mutants. Flag-tagged EGR1-FL and HA-tagged TET1 deletion mutants as shown in the schematic illustration were co-expressed in HEK293T cells. **d** Interactions between TET1s-CD and EGR1 deletion mutants. HA-tagged TET1-CD and Flag-tagged Egr1 deletion mutants as shown in the schematic illustration were co-expressed in HEK293T cells. Protein-protein interactions were examined by IP-western blot using the antibodies indicated. Source data are provided as a Source Data file

The expression of mouse full length TET1 protein (2,039 aa, ~220 KDa) is restricted to early embryonic and primordial germ cells, and a short isoform TET1s (residues 654–2,039, ~170 KDa) is expressed in somatic tissues including brain^37^. To further determine whether TET1 may participate in the demethylation of EGR1 binding sites, we performed co-immunoprecipitation assays (Co-IP) using mouse frontal cortices. The short isoform TET1s was found in the EGR1 immunopreciated complex (Fig. 2a) and EGR1 could be precipitated together with TET1s (Fig. 2b). To narrow down the binding regions responsible for the EGR1-TET1s interaction, we first conducted Co-IP in HEK293T cells with Flag-tagged EGR1 co-transfected with HA-tagged TET1s-N (residues 654–1,366) or TET1s-CD (residues 1,367–2,039), respectively. Using anti-HA antibody to probe anti-Flag immunoprecipitates, we found EGR1 binds to TET1s-CD, but not TET1s-N (Fig. 2c). On the other hand, the immunoblotting analyses of anti-Flag immunoprecipitates for Flag-tagged EGR1 truncated proteins demonstrated that TET1s-CD binds to EGR1-C (residues 318–533) but not EGR1-N (residues 1–318) (Fig. 2d). To further pinpoint the regions that mediate their interaction, we removed 171 aa from TET1s-CD to delete the cystine-rich domain (TET1s-CD∆c, residues 1,538–2,039) and 81 aa from EGR1-FL to delete the zinc finger domain (EGR1∆z, residues 1–335, 417–533). EGR1∆z interacted with TET1s-CD but TET1s-CD∆c lost the interaction capacity with EGR1 (Supplementary Fig. 9a & b). Additionally, the EGR1-TET1s interaction was preserved with the presence of ethidium bromide (Supplementary Fig. 9c). This result suggests EGR1-TET1s interaction is DNA-independent, which is consistent with the observation that the removal of zinc finger domain from EGR1 does not interfere with such an interaction. Altogether, these data demonstrate that EGR1 and TET1s form a complex mediated by the C-terminal regions of both proteins.

**Fig. 3:**
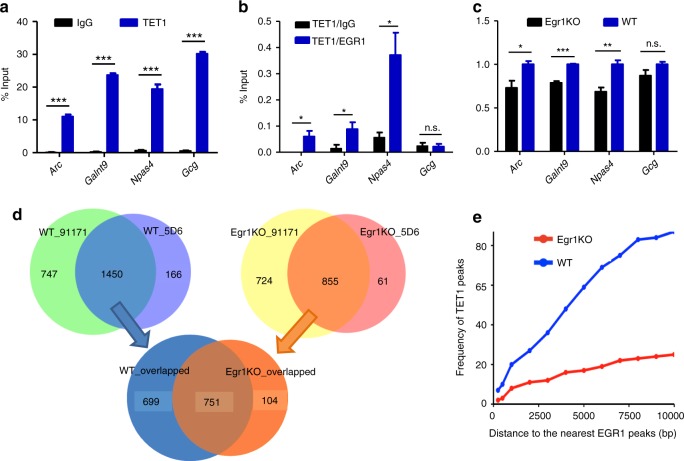
EGR1 recruits TET1 to its target sites. **a** TET1 ChIP-qPCR assay in wild-type mouse frontal cortices. **b** Sequential ChIP-qPCR assay in wild-type mouse frontal cortices. The first antibody used was anti-TET1; the secondary antibody used was anti-EGR1 and normal rabbit IgG. *Gcg* locus serves as negative control for EGR1 binding. **c** ChIP-qPCR assay in frontal cortices of Egr1KO and wild-type mice. TET1 enrichment is normalized to the enrichment in WT. *P*-values were calculated with *t*-test, *P < 0.05, **P < 0.01, ***P < 0.001. n.s., not significant. Error bars ± standard deviation (s.d.) from three technical replicates. **d** Venn diagrams show the overlapped TET1 peaks generated with two distinct antibodies (91171 and 5D6, Active Motif) or from WT and Egr1KO frontal cortices. **e** The distribution of TET1 peaks relatively to their nearest EGR1 peaks. The distance of TET1 peak to its nearest EGR1 peak refers to the number of nucleotides between the centers of two peaks

We next sought to investigate whether EGR1 recruits TET1s to its binding sites. For three EGR1 binding sites, we performed ChIP-qPCR analysis using frontal cortices from wild-type mice and observed the co-occupancy of EGR1 and TET1s (Fig. 3a and Supplementary Fig. 4a). To rule out the possibility that the concurrent enrichments may result from cell population heterogeneity, we conducted sequential ChIP-qPCR and confirmed EGR1 and TET1s indeed present at these loci simultaneously (Fig. 3b). In addition, we examined whether the depletion of EGR1 may affect the occupancy of TET1s at these loci. ChIP-qPCR assays showed TET1s enrichment on these loci was significantly decreased in the frontal cortices derived from *Egr1* knockout mice (Egr1KO) compared with those from wild-type mice (Fig. 3c). We expanded the study to perform TET1 ChIP-seq for the frontal cortices derived from Egr1KO and WT mice using two validated anti-TET1 antibodies (Supplementary Data 3). The libraries generated for two biological replicates and with the two antibodies yielded peaks that largely overlapped (Supplementary Fig. 10 and Fig. 3d). A total number of 855 and 1,450 TET1 peaks were determined for the frontal cortices of Egr1KO and WT mice respectively (Supplementary Data 3), with four peaks validated by independent ChIP-qPCR assays (Supplementary Fig. 10e, f). Approximately 87.8% (751 out of 855) TET1 peaks identified in Egr1KO mice overlap with those identified in WT mice. Only eight EGR1 peaks were found to overlap with these 751 TET1 peaks and no EGR1 peak overlaps with the rest 104 peaks identified in Egr1KO only. However, 61 EGR1 peaks overlap with the 699 TET1 peaks identified in WT but not in Egr1KO. GO terms including “axon guidance” and “cell morphogenesis involved in neuron differentiation” were enriched for genes associated with TET1 peaks identified in WT mice but not in Egr1KO mice (Supplementary Fig. 11). We further examined the influence of *Egr1* loss on the distribution of TET1 peaks. Compared to those in WT control, TET1 peaks in Egr1KO mice shift away from their adjacent EGR1 peaks (Fig. 3e). Altogether, these results indicate that EGR1 may attract TET1 to genomic regions flanking EGR1 binding sites.

### EGR1 coordinates with TET1 in gene expression regulation

**Fig. 4:**
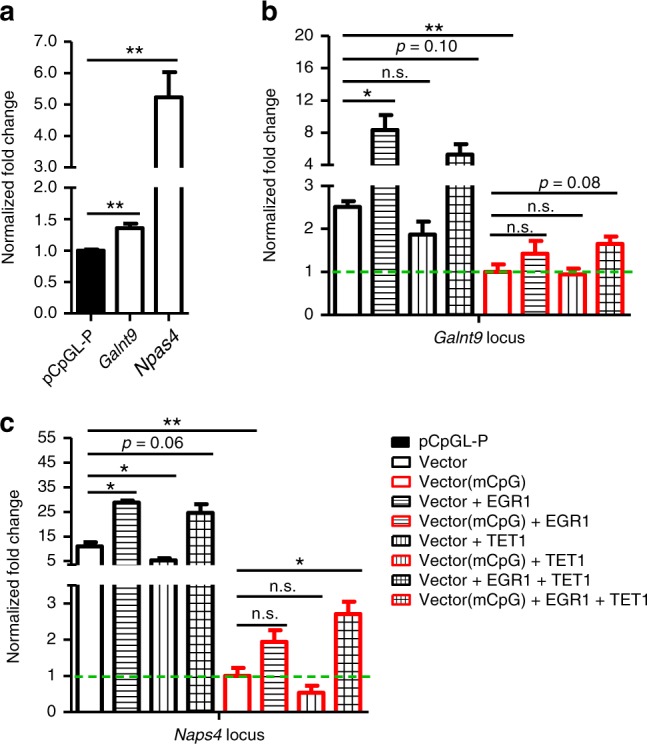
Cooperativity of EGR1 and TET1 modulate the enhancer activity of EGR1 binding sites. **a** Luciferase reporter assays for the control vector pCpGL-P and constructs with *Galnt9*, *Npas4* locus. Fold change was normalized to the control vector pCpGL-P. Luciferase reporter assays for unmethylated or methylated *Galnt9* (**b**) and *Npas4* (**c**) constructs under either *Egr1*/*Tet1* singularly or co-expression in primary cortical neurons. In figure **b**–**c**, fold changes were normalized to the methylated vectors without *Egr1/Tet1* overexpression. Luciferase activity was measured at 48 h after transfection and normalized against the activity of a co-transfected firefly construct. mCpG represents methylated constructs. *P*-values were determined by *t*-test, *P < 0.05, **P < 0.01. Values represent mean ± s.d. from three biological replicates

We examined whether the interaction between EGR1 and TET1 would have epigenetic regulatory effects. We started with two loci within *Galnt9* and *Npas4* genes, which were identified to be EGR1 binding sites by our ChIP-seq and validated by ChIP-qPCR to be enriched for H3K27ac enhancer mark (Supplementary Fig. 4c). To test their enhancer activities in primary culture of cortical neurons (Supplementary Fig. 12), the genomic fragments were cloned to the upstream of EF1 promoter in the pCpG-free vector, respectively. Compared with the control vector, *Npas4* and *Galnt9* loci significantly promoted the gene expression from the basal EF1 promoter in the enhancer luciferase assays (Fig. 4a). To further examine whether their enhancer activities are under epigenetic control, prior to transfection, the constructs containing two loci were methylated in vitro with CpG methyltransferase, M.SssI. The methylation of these loci greatly reduced their enhancer activities (Fig. 4b–c). We utilized the unmethylated and methylated reporter constructs to examine their enhancer activities with or without EGR1 or/and TET1 overexpression. For unmethylated reporter constructs, EGR1 overexpression alone could significantly increase luciferase signals of reporters, consistent with the fact that EGR1 as a transcriptional factor can induce the enhancer activities of its binding sites. By contrast, TET1 overexpression alone displayed no increase in luciferase signal of these reporter vectors (Fig. 4b–c). For methylated reporter constructs, we observed increases in the enhancer activities when EGR1 was co-overexpressed with TET1 (*p* = 0.01 for *Npas4* and *p* = 0.08 for *Galnt9*). This result suggests that EGR1 and TET1 may cooperate to activate the expression of EGR1 downstream gene by DNA demethylation.

To further investigate the epigenetic effect of EGR1 and TET1 on their binding sites, we examined the methylation statuses of *Galnt9* and *Npas4* loci in primary neurons with *Egr1* or/and *Tet1* knockdown. Primary mouse cortical neurons isolated at E16.5 was adopted as in vitro neuronal activity model for KCl depolarization^38,39^. Western blot showed that EGR1 increased dramatically upon neuronal activation, while TET1 protein level remains relatively constant (Supplementary Fig. 13a). Importantly, methylation level of *Galnt9* and *Npas4* loci significantly reduced in E16.5 cortical neurons upon KCl stimulation (Supplementary Fig. 13b). To explore whether *Egr1* and *Tet1* mediate methylation changes at these two loci, *Egr1* and *Tet1* were knocked down with their corresponding shRNA constructs (Supplementary Fig. 13c). Pyrosequencing results for bisulfite converted DNA indicated that *Egr1/Tet1* shRNA knockdown or the double knockdown significantly increased their methylation levels (Supplementary Fig. 13d). The RT-qPCR results showed that the mRNA level of *Galnt9* was reduced with *Egr1* knockdown, and significantly lower with *Egr1* and *Tet1* double knockdown. However, no significant change in *Npas4* mRNA level was observed (Supplementary Fig. 13e). This may be due to the incomplete knockdown of *Egr1*/*Tet1* and the subtle methylation changes (~3%) achieved on *Npas4* locus over a short period of stimulation. Collectively, these data suggest that EGR1 is able to coordinate with TET1 to epigenetically regulate its target loci but the significant alteration in expression of some downstream genes may require repeated stimulations over a long period of time.

### EGR1KO and TET1KO mice share aberrant methylation profiles

It has been documented that Egr1KO mice show impaired long-term memory^38^. Recent studies show *Tet1* knockout mice (Tet1KO) exhibited significant deficiency in memory retention^12^, abnormal long-term depression and impaired memory extinction^9^. To examine the epigenetic effects of *Egr1* or *Tet1* loss, we performed methylome and transcriptome analyses for the frontal cortices derived from Egr1KO and Tet1KO mice. The genotypes of Egr1KO and Tet1KO mice were confirmed by the read coverage along *Egr1* and *Tet1* loci (Supplementary Fig. 14). Since EGR1 binding sites are enriched in promoters and CG rich regions, we performed reduced representation bisulfite sequencing using restriction enzymes MseI and MluCI to remove AT-rich regions. For four methylomes, we generated 211 to 287 million read pairs with an average of 140 million read pairs uniquely mapped to mouse reference genome (Supplementary Data 4). On average, we obtained methylation information for 48.9% of all CpG dinucleotides in the mouse genome and 18.1% of all CpG sites covered by at least 10 reads. Based on spiked-in unmethylated λ DNA control, the bisulfite conversion rates for four libraries were determined as 99.0% on average.

We observed strong correlations between biological replicates for two Egr1KO mice and two Tet1KO mice, respectively (Supplementary Fig. 15). For the corresponding four transcriptomes, we generated 39 million read pairs on average, 86.0% of which were unambiguously mapped (Supplementary Data 4). We determined pairwise Pearson’s correlation at gene expression level and validated the consistency of RNA-seq results for biological replicates (Supplementary Fig. 16). Compared with the methylome of the frontal cortex from wild-type mice, we identified 49,991 differentially methylated sites (DMSs) in Egr1KO with 34,747 (69.5%) hypermethylated and 15,244 (30.5%) hypomethylated, and 113,488 DMSs in Tet1KO with 94,862 (83.6%) hypermethylated and 18,626 (16.4%) hypomethylated. To examine the association between EGR1 binding and methylation changes in KO mice, we determined the distribution of DMSs at the flanking of EGR1 binding sites. The density of hypermethylated DMSs in Egr1KO increases when approaching to the centers of EGR1 peaks, while hypomethylated DMSs in both Egr1KO and Tet1KO are depleted from EGR1 peaks (Supplementary Fig. 17a). When DMSs were clustered into differentially methylated regions (DMRs), the increased enrichment in EGR1 peaks was observed for hypermethylated DMRs from both Egr1KO and Tet1KO mice (Supplementary Fig. 17b). We next focused on the aforementioned 1,925 EGR1 binding sites, which display methylation loss from d0 to 6 weeks. Approximately 83.0% and 84.5% of these loci show increased methylation in KO mice; particularly, 19.4% and 24.7% loci are with hypermethylated DMSs in Egr1KO and Tet1KO mice respectively. These results indicate that EGR1 and TET1 are indispensable for the demethylation of some EGR1 binding sites during brain development.

**Fig. 5:**
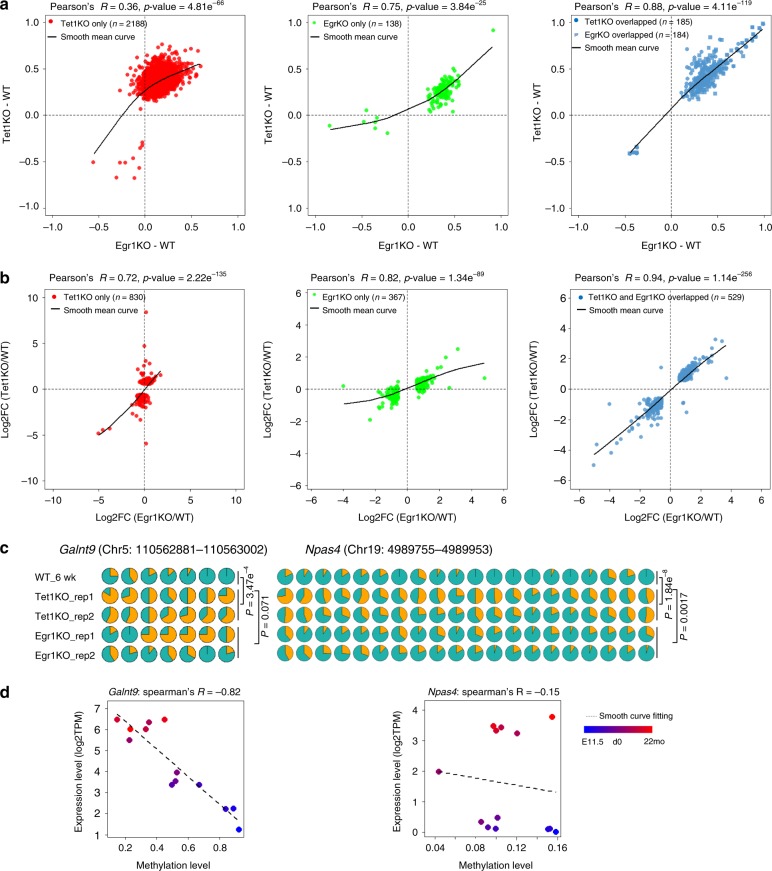
Correlations of DNA methylation and gene expression profiles between Egr1KO and Tet1KO frontal cortices. Methylation correlations (**a**) and gene expression correlations (**b**) between Egr1KO and Tet1KO mice. **c** Aberrant DNA methylation on *Galnt9* and *Npas4* loci. Each CpG is represented by a circle; yellow in circles indicates the percentage of methylation in each CpG site. The statistical significance of methylation differences between Egr1/Tet1KO and WT mice was evaluated with the Wilcoxon rank-sum test. **d** The correlations between DNA methylation levels of *Galnt9, Npas4* loci and corresponding gene expression during brain development from embryonic day 11.5 (E11.5) (denoted in blue color) to 22 months (22 mo) (denoted in red color)

Compared to wild-type mice, 322 and 2,373 DMRs were identified in the frontal cortices of Egr1KO and Tet1KO mice respectively and these DMRs are significantly overlapped (Hypergeometric test, *p*-value = 8.36e-14). In addition, the methylation correlation of the overlapping 184 DMRs between Egr1KO and Tet1KO is 0.88 (Pearson’s *r*). The knockout of *Tet1* has a broader and more severe impact on the methylomes compared to the loss of *Egr1*. Intriguingly, for DMRs identified in Tet1KO mice only, moderate changes in methylation were often observed in Egr1KO mice as well, and vice versa (Fig. 5a). We further divided DMRs into hypermethylated or hypomethylated in either Egr1KO or Tet1KO and obtained their methylation profiles across developmental stages and in neuronal cell types (Supplementary Fig. 18). Methylation loss during development and in neurons was observed for around 78.0% and 56.2% of DMRs identified in Egr1KO and Tet1KO mice, respectively. Interestingly, 9.1% Egr1KO DMRs and 13.9% Tet1KO DMRs were found to be constantly hypomethylated across developmental stages but with increased methylation in KO mice. This result suggests EGR1 and TET1 are required for the maintenance of demethylation statues for some genomic loci, which are not or lowly methylated since early brain development (at E11.5 or earlier).

Compared to wild-type controls, 896 and 1,359 differentially expressed genes (DEGs) were determined in Egr1KO and Tet1KO mice respectively with 529 of them shared (Fig. 5b). Similar to what observed for methylomes, for these 529 genes, the correlation in the expression changes *vs* wild type is 0.94 (*p*-value = 1.14e-256) between Tet1KO mice and Egr1KO mice. For all DEGs identified either in Egr1KO or Tet1KO mice, strong correlations were observed between the two kinds of KO mice. GO annotation analyses showed that both Egr1KO and Tet1KO DEGs are involved in several biological processes (BPs) related to central nervous system development or neural tube development, including potassium ion transport and Notch signaling pathway which plays critical roles in brain development^39^ (Supplementary Fig. 19 and Supplementary Data 5). To explore the relationship between DMR methylation and gene expression, we calculated the Spearman’s correlation coefficients of hypermethylated DMRs identified in Egr1KO or Tet1KO mice (Supplementary Fig. 20). Negative correlations between methylation level and gene expression were observed for DMRs in 5′-UTR, Promoter and Distal Promoters. In addition, significant increases in methylation were observed in KO mice for the three EGR1 binding sites within *Galnt9* and *Npas4* genes (Fig. 5c). The methylation levels of these loci, *Galnt9* in particular, are negatively correlated with gene expression (Fig. 5d).

Lastly, hypermethylated DMRs identified in either Egr1KO or Tet1KO show low methylation in excitatory neurons compared with PV and VIP neurons (Supplementary Fig. 18). This prompts us to make use of single-cell brain methylome data^40^ for additional bioinformatics analyses on the cell-subtype-specific function of EGR1 bindings. We confirmed that the hypermethylated DMSs identified in Egr1KO mice are significantly enriched on excitatory-neuron-specific hypomethylated regions determined in single cell analyses (Supplementary Fig. 21), especially for excitatory-neuron-subtype mL5-1 (odds ratio = 1.4, Binomial test, *p*-value = 4.8e-42). EGR1 binding sites are significantly enriched on excitatory-neuron-specific hypomethylated regions but excluded from inhibitory-neuron-specific ones (Supplementary Fig. 22). For instance, the enrichment of EGR1 binding sites on hypomethylated regions in excitatory-neuronal subtype mL4 is highly significant (odds ratio: 1.7, Binomial test, *p*-value = 6.7e-83). In addition, the enrichment of EGR1 binding is correlated with the bindings of other early response genes including TFs induced by neuronal activity, such as JUNB, FOSB, CFOS, and NPAS4 (Supplementary Fig. 23).

## Discussion

The link between epigenetic changes and neuronal activity has been well established, together with the gradual recognition of critical roles of TET DNA demethylases in learning and memory^6–9^. Apparently, epigenetic changes upon neuronal activation are not random but TET enzymes do not display DNA binding specificity. Our study shows how TET1 gains its specificity *via* the interaction with EGR1, a sequence specific DNA binding protein. On the other hand, as a key member of immediate early genes, *Egr1* has been known for decades to play an essential role in transcriptional response to environmental stimuli. *Egr1* is an important mediator of the effects of early-life experience and directly regulate genes controlling synaptic plasticity in both physiological and pathological conditions^1,20,23,38^. *Egr1* expression has been widely used as a marker for neuronal activation but how it leaves memory trace remains elusive. In this study, we provided a key piece of evidence that may help in solving this puzzle at the epigenetic level. Although neurogenesis and neuronal migration are largely completed at birth in mouse, postnatal brains continue forming synapses and neural circuits and undergo activity-dependent refinements. *Egr1* has been shown to control newborn neuron selection and maturation during the critical period of a few weeks after birth^21^. The decoding of epigenetic machineries during this developmental period is critical for a complete understanding of the mechanisms that underlie late-stage refinement of maturing neuronal circuits. Of note, *Egr1* gene continues to have functions in the adult brain and may have pathological significance in Alzheimer’s disease^41^.

**Fig. 6:**
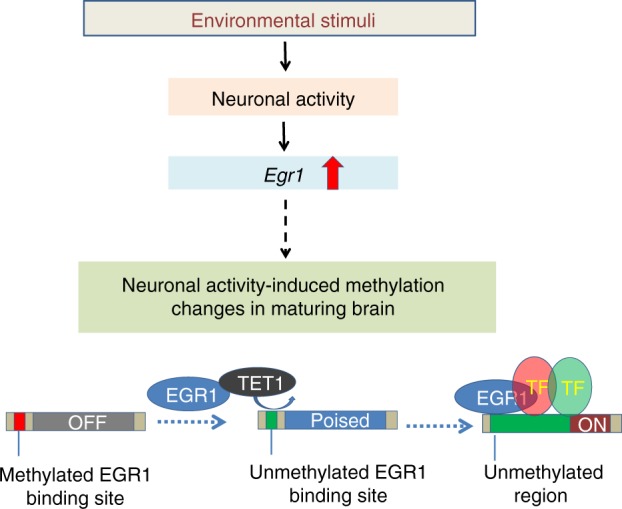
A simplified model for EGR1 and TET1 interaction linking environmental stimuli to brain methylome programming. At birth, *Egr1*-mediated and neuronal activity-induced genes are silenced with methylated EGR1 binding sites. During postnatal development and upon neuronal activity, the increase in expression of *Tet1* and *Egr1* leads to the demethylation of EGR1 binding sites to facilitate the binding of co-factors and shifts the genes to either “Poised” or “ON” states. DNA methylation cannot block EGR1 binding but may prevent the bindings of other transcription factors, which bind to the regions adjacent to EGR1 binding sites. Thus, the demethylation of EGR1 binding sites may facilitate the formation of stronger transcription enhanceosomes

Our study provides several key evidences for the interaction between EGR1 and TET1. First, our results reveal that extensive DNA demethylation occurs in thousands of EGR1 binding sites during the postnatal frontal cortex development. Second, the C-terminals of EGR1 and TET1 are required for their interaction. The co-occupancy of EGR1 and TET1 at target loci were confirmed with sequential ChIP analyses. In the presence of EGR1, TET1 is capable to achieve locus-specific demethylation and activate the expression of EGR1 downstream genes. Third, both EGR1 and TET1 are indispensable for the demethylation of a common set of EGR1 binding sites that show aberrant DNA methylation in Egr1KO and Tet1KO mice. Altogether, our data support a model that links environmental stimuli to brain methylome programming (Fig. 6). At birth, a subset of *Egr1*-mediated and neuronal activity-induced genes are silenced with methylated EGR1 binding sites. During early postnatal development, the overexpression of *Tet1* and *Egr1* upon neuronal activation demethylate EGR1 binding sites and shift the genes to either “Poised” or “ON” states. DNA methylation cannot block EGR1 binding but may prevent the bindings of other TFs, which bind to the regions adjacent to EGR1 binding sites. Thus, the demethylation of EGR1 binding sites may facilitate the formation of stronger transcription enhanceosomes.

Our study raises a few interesting questions. Could EGR and TET interaction become a general mechanism for various kinds of cells to keep epigenetic memory in response to stimuli? EGR family members are involved in a variety of BPs and the epigenetic memory may not be limited to the nervous system. For instance, *Egr1, Egr2 and Egr3* have been shown to be critical for the response to external signals and to direct lineage differentiation in immune system^42,43^. More dedicated study is needed to fully address how TET and EGR family members may interact with each other and to understand their combinatorial interactions in various BPs. In our study, we found Egr1KO and Tet1KO have significant effects but limited to less than 30% of the 1,925 EGR1 binding sites which show demethylation during postnatal brain development. Compared with the loss of *Egr1*, the *Tet1* knockout has a much more severe outcome and broader impact in epigenome. These results suggest a compensatory mechanism to rescue the loss of *Egr1* or *Tet1* in the frontal cortex, and TET1 may have other partners to recognize genomic loci where EGR1 can bind. Since EGR1 is a neuronal activity induced TF, EGR1 protein level is very low in neurons at rest. TET1 protein levels remains relatively constant before and after KCl stimulation. This suggests the majority of TET1 proteins are not necessarily bound by EGR1 at all times. Even with the massive EGR1 expression pattern in activated neurons, the interaction between TET1 and EGR1 could be transient. As reflected in our TET1 ChIP-seq result, 51.8% of TET1 peaks identified in WT mice were present in Egr1KO mice. In this study, we found that the genome distribution of TET1s binding in brain is biased toward intergenic regions, compared to those full length TET1 binding sites reported in mouse embryonic stem cells^37,44,45^. The short isoform TET1s expressed in mouse brain lacks CXXC and BC domain (“before CXXC”)^37^. The CXXC domain helps the binding of full length TET1 to CpG rich regions while the BC domain assists in its global chromatin affinity. Thus, TET1s shows low chromatin affinity and increased presence in soluble fraction instead of chromatin^37^. Our finding is also consistent with a recent study with cancer cells that the full length TET1 protects CpG islands from methylation while TET1s mediates demethylation outside CpG islands and the recruitment of TET1s to chromatin likely needs specific factors^46^. Additional effort is required to identify other proteins associated with TET1s to gain a better understanding on how it functions in brain. Lastly, the integrated bioinformatics analyses with single neuron methylomes suggest the methylation changes of EGR1 binding sites are largely restricted to a subset of excitatory neurons. It would be interesting to explore whether different kinds of neurons would adopt distinct epigenetic programming mechanisms in future.

## Methods

### Animals

All animal experiments were performed according to guidelines of the Institutional Animal Care and Use Committee at Virginia Tech (Blacksburg, VA, USA). The *Egr1* heterogeneous mouse strain (B6N; 129-*Egr1*^tm1Jmi/J^), the *Tet1* heterogeneous mouse strain (B6; 129S4-*Tet1*^tm1.1Jae/J^) were purchased from The Jackson Laboratory. Genomic DNA was isolated from tail biopsies and genotyped by PCR according to The Jackson Laboratory’s protocols.

### E16.5 mouse cortical neurons and HEK293T culture

Primary mouse cortical neuron cultures were prepared from E16.5 C57BL/6 embryonic mouse cortices as described previously^47^ with slight modifications. Briefly, E16.5 C57BL/6 mouse embryo cortices were dissected and then dissociated into single-cell suspension by enzymatic digestion using Neural tissue dissociation kit (P) (Cat# 130-092-628) with gentleMACS Octo Dissociator with Heaters (Cat# 130-096-427) from Miltenyi Biotech according to the manufacturer’s protocol. After dissociation, neurons were seeded at an approximate density of 4 × 10^7^ on 15-cm dishes. The dishes were pre-coated with poly-ornithine (20 μg ml^−1^, Sigma) and mouse laminin (4 μg/mL, Invitrogen) at 37 °C for 2 h, washed three times with sterile water and then once with Neurobasal Medium (Life Technologies) before use. Neurons were grown in 30 ml neuronal medium consisting of Neurobasal medium containing B27 supplement (2%; Invitrogen), penicillin-streptomycin (1%, ThermoFisher) and Glutamax (1%, ThermoFisher). Neurons were then placed in a cell culture incubator that maintained a temperature of 37 °C and a CO_2_ concentration of 5%. Two hours after plating neurons, the medium was completely aspirated and replaced with fresh warm neuronal medium. Neurons were grown in vitro for 7 days. Ten milliliters of the medium was replaced with 12 ml fresh warm medium on DIV3 and DIV6. Prior to KCl depolarization, DIV6 neurons were silenced overnight with 1 μM tetrodotoxin (TTX; Fisher) and 100 μM DL-2-amino-5-phosphopentanoic acid (DL-AP5; Fisher). The next day neurons were stimulated with 55 mM KCl and harvested at desired time points.

HEK293T (ATCC, CRL-11268™) cells were maintained in Dulbecco’s Modified Eagle’s Medium (DMEM) (Life Technologies) supplemented with 10% heat-inactivated fetal bovine serum (Corning) and 1% penicillin/streptomycin (Gibco). All cells were cultured at 37 °C in a humidified atmosphere of 5% CO_2_ incubator.

### Immunocytochemistry

E16.5 mouse cortical neurons were seeded on 8-well chamber and cultured in vitro for 7 days (DIV7). The neurons were rinsed once with PBS and fixed with 4% paraformaldehyde in PBS for 15 min at room temperature. After washing three times with PBS, cells were permeabilized with 0.2% TritonX-100 in PBS for 10 min. Cells were then washed three times with PBS, blocked with 5% Normal Goat Serum (ThermoFisher) in 1×PBS for RT for 1 h, followed by incubation with primary antibodies at 4 °C overnight. After washing three times with 1×PBS, cells were incubated with corresponding Cy3 conjugated anti-rabbit IgG (A10520, Invitrogen), Alexa Fluor 488 conjugated anti-mouse IgG (A10680, Invitrogen) secondary antibody at RT in darkness for 1 h. After washing 3 × 5 min with 1×PBS, cells were then mounted with DAPI-Fluoromount-G™ Clear Mounting Media (SouthernBiotech, 010020). Fluorescent images were acquired using immunofluorescence microscope.

### Antibodies

Rabbit anti-TET1 (Millipore, 09–872), mouse anti-TET1 (Active Motif, 91171), rat anti-TET1 (Active Motif, 61741, refer to as 5D6), mouse anti-HA (Invitrogen, 26183), mouse anti-Flag (sigma, F1804 and F7425), mouse anti-Tuj1 (Biolegend, 801201), rabbit anti-GFAP (Sigma, HPA056030) antibodies were purchased commercially. Rabbit anti-EGR1 antibody (sc-189), mouse anti-EGR1 antibody (sc-101033), rabbit normal IgG (sc-2027) and mouse normal IgG (sc-2025) were purchased from Santa Cruz Biotechnology. For western blot analysis, goat anti-rabbit horseradish peroxidase-conjugated secondary antibody (Invitrogen, 65–6120) was used at a 1:5000 dilution, goat anti-mouse horseradish peroxidase-conjugated secondary antibody (Sigma, A8924) was used at a 1:10,000 dilution. The antibodies used for the IP and western blot experiments were summarized in Supplementary Data 6.

### Plasmid construction

Flag-tagged mouse EGR1 expression vector was obtained from Addgene (plasmid 11729). Plasmids encoding Flag-tagged EGR1 N-terminal (amino acids 1–318), EGR1 C-terminal (amino acids 318–533), EGR1∆z (amino acids 1–335, 417–533) were generated by subcloning of the DNA fragments into EcoRI and XhoI sites of pcDNA3 vector. In order to clone truncated versions of TET1 protein, cDNA was synthesized from mRNA of C57BL/6J mice. Plasmids encoding HA-tagged TET1s-FL (amino acids 654–2039), TET1s-N (amino acids 654–1366), TET1s-CD domain (amino acids 1367–2039), TET1s-CD∆c (amino acids 1538–2039) were generated by subcloning of the DNA fragments into BamHI and XbaI of pcDNA3 vector.

For shRNA cloning, pLKO.1 puro, a gift from Bob Weinberg (MIT) (Addgene plasmid # 8453) was digested with AgeI and EcoRI for 4 h at 37 °C. Digested plasmid was excised from the gel and purified with GeneJET™ gel extraction kit (Thermo Fisher). Oligos were designed for the following target sequences and annealed (*Tet1* shRNA GCTCATGGAGACTAGGTTTGG, *Egr1* shRNA AGCGCTAGACCATCAAGTT) as described previously^7,48^, Annealed oligos were ligated into the digested vector with T4 ligase (NEB); and colonies were screened by sequencing. Scramble shRNA was a gift from David Sabatini (MIT) (Addgene plasmid # 1864).

For luciferase constructs, two selected EGR1 binding loci: *Galnt9* and *Npas4* loci were amplified from genomic DNA of C57BL/6 mice using the primers listed in Supplementary Data 7. After enzymatic digestion for at least 4 h, PCR-amplified products were cloned into pCpGfree-promoter-Lucia Vector (Invivogen). All inserts were verified by Sanger sequencing.

### Lentivirus generation and transfection

Dishes were plated with 50 μg/mL poly-D-lysine (Sigma) and HEK293T cells (ATCC® CRL-3216™) were plated at 70–80% confluent before transfection. Lentiviral helpers and shRNA constructs were transfected at a 1:3 ratio using X-tremeGENE 9™ (Roche) according to the manufacturer’s instructions. Media was changed at 24 h after transfection. Infectious media containing virus was collected 40 h later and filtered with a 0.45uM PES membrane filter (Millipore). Viral media was concentrated at 100,000 × *g* for 90 min at 4 °C (Beckman Optima Max tabletop ultracentrifuge). Viral pellets were resuspended in sterile PBS overnight at 4 °C. Lentiviral particles (IU/mL) were determined by real-time qPCR (BioRad CFX96) using the qPCR Lentivirus Titration(Titer) Kit (ABM) according to the manufacturer’s instructions using iTaq™ Universal SYBR® Green Supermix (BioRad). Lentivirus particles were added to cortical neuronal culture at DIV5 with final concentration of 1.0 × 10^6^ IU/mL. After incubation for 24 h, the medium was replaced with fresh neuronal culture medium and continue culture for another 24 h prior to KCl stimulation.

### Luciferase reporter assays

Luciferase reporter constructs were either mock-treated or methylated in vitro with M.SssI methylase (NEB) for at least 4 h at 37 °C and purified with PureLink PCR Purification Kit (Qiagen). E16.5 mouse cortical neurons were seeded at 3 × 10^5^/well in 24-well plates overnight, then transfected with 0.3 μg of reporter constructs and 0.02 μg of firefly luciferase control vector pGL 4.13 (Promega) using Lipofectamine 3000 (Invitrogen). For each sample, triplicate transfections were carried out. 48 h after transfection, cell lysates and the medium were assayed for luciferase activity by Dual Luciferase Reporter Assay (Promega). Lucia luciferase activity of individual transfections was normalized to firefly luciferase activity and analyzed relatively to empty pCpG-free promoter vector^49^.

### qRT-PCR analysis

Total RNA was extracted using the RNeasy kit (Qiagen) and cDNA was generated using a high-capacity cDNA reverse transcription kit (Applied Biosystems). qRT-PCR experiments were performed using GoTaq® qPCR Master Mix (Promega) on StepOnePlus™ Real-Time PCR Systems. Relative expression levels were determined by comparative ∆∆Ct method with beta-actin as an internal reference control.

### Co-immunoprecipitation and western blotting

HEK293T cells were transfected with plasmids by Lipofectamine 3000 (Invitrogen) according to the manufacturer’s instructions. Cell lysis, immunoprecipitation, and western blot analysis was performed as previously described^50^. Briefly, cells were lysed in ice-cold lysis buffer (50 mM Tris-HCl (pH 7.4), 150 mM NaCl, 1% Triton X-100, 2 mM EDTA), supplemented with protease inhibitor cocktail (Thermo scientific). Immunoprecipitation was carried out by incubating specific antibody coupled Dynabeads Protein A or Protein G (Life Technologies) at 4 °C overnight. The samples were washed four times with ice-cold lysis buffer, and then suspended in 30 μl loading buffer (Life Technologies). After boiling at 95 °C for 5 min, the samples were analyzed by western blot with specific antibodies.

### ChIP-seq, ChIP-qPCR, and sequential ChIP-qPCR

Frontal cortices of adult mice (6-week old) were dissected on ice, cross-linked with 1% formaldehyde, and then neutrolized by 0.125 M glycine. Samples were lysed in lysis buffer I (50 mM HEPES-KOH, 1 mM EDTA, pH 8.0, 140 mM NaCl, 0.25% Triton X-100, 0.5% NP-40 and 10% glycerol and halt protease inhibitor cocktail), lysis buffer II (10 mM Tris-HCl, pH 8.0, 1 mM EDTA, pH 8.0, 200 mM NaCl and 0.5 mM EGTA, pH 8.0 and halt protease inhibitor cocktail), respectively. Samples were then sonicated with Covaris M2 (Covaris) into 200–700 bp. Ten percent of pre-cleared chromatin was stored as input material. The rest was incubated overnight at 4 °C with 30 μl of Dynal protein A/G magnetic beads (Life technologies) that had been pre-incubated with specific antibodies.

For EGR1 ChIP-seq, frontal cortices from 4–5 male mice at 6-week-old were used for a single ChIP experiment. The genomic DNA fragments were obtained from anti-EGR1 immunoprecipitated chromatin as mentioned above, and library construction was performed as described previously^51^. DNA in the range 270–600 bp was recovered by Pippin Prep (Sage Science), after size distribution assessment by Agilent bioanalyzer and quantification by qPCR (Kapa Library quantification kit), libraries were subjected to 100-bp paired-end read sequencing on the Illumina HiSeq 2000 platform.

For TET1 ChIP-seq, the genotype of Egr1WT and Egr1KO mice were first confirmed by genotyping PCR and western blot. Frontal cortices from two male mice at 6-week-old were used for a single ChIP experiment. The genomic DNA fragments were obtained from anti-TET1 immunoprecipitated chromatin as mentioned above, and the library construction was performed as described above. The libraries were subjected to 150-bp paired-end read sequencing on the Illumina HiSeq 4000 platform.

For ChIP-qPCR, frontal cortices from two male mice at 6-week-old were used for a single ChIP experiment. Real-time PCR was performed using GoTaq® qPCR Master Mix (Promega) on StepOnePlus™ Real-Time PCR Systems. Antibodies used for immunoprecipitation were anti-H3K27ac (ab4729, Abcam), anti-EGR1 (sc-189, Santa Cruz Biotechnology), anti-TET1 (09–872, Millipore), anti-TET1 (61741, Active Motif) and normal rabbit IgG (sc-2027, Santa Cruz Biotechnology). Primers specific for chosen genomic regions were summarized in Supplementary Data 7.

For sequential ChIP-qPCR, frontal cortices from two wild-type male mice at 6-week-old were used for a single sequential ChIP experiment. Chromatin was prepared as described above, after chromatin immunoprecipitation with the first antibody, magnetic beads were resuspended in 1% SDS supplemented with 10 mM DTT at 37 °C for 30 min. The eluate was then diluted 20 times with dilution buffer (1% Triton X-100, 2 mM EDTA, 20 mM Tris-HCl pH8, 150 mM NaCl) and the second antibody was added for immunoprecipitation overnight. Beads were then washed, reverse crosslinked and DNA purified. The antibodies used were anti-EGR1 (sc-189, Santa Cruz Biotechnology), anti-TET1 (09–872, Millipore) and normal rabbit IgG (sc-2027, Santa Cruz Biotechnology).

### ChIP-seq data analysis

All reads for ChIPseq libraries were first trimmed according to their sequencing qualities, then the trimmed reads were mapped to the mouse reference (mm10) by using Bowtie^52^ with parameters “–n 2 –l 50”. EGR1 Peak calling was performed using SPP^53^ with parameters “-npeak = 300000 -p = 5 -savr -savp –rf”. The data reproducibility between biological replicates was examined following irreproducible discovery rate (IDR) framework with parameters “0 F signal.value”^54^. The IDR threshold was set as 2%, which is recommended by ENCODE. The reported peaks were produced by merging highly reproducible peaks of biological replicates. The top 200 most significant peaks were selected for de novo motif discovery using MEME Suite^55^. Known motif enrichment analysis was performed using the script findMotifs.pl in HOMER with parameter “–mset vertebrates”. TET1 peaks were determined with MACS2 using broad parameters including the cutoff for fold change as 2 and the cutoff for *q* value as 1E-5.

### Methylome analysis to identify bipolar methylated loci

The bipolar DNA methylation inference was performed by pooling all human and mouse brain methylome datasets together, respectively, and then bipolar DNA methylation loci with at least 100Xs read coverage were identified following the procedure described previously^29^. After merging of the overlapped loci, a total of 39,114 and 21,946 bipolar DNA methylation loci were identified for human and mouse brain methylomes, respectively.

### RRBS library construction and data analysis

Genomic DNA from mouse frontal cortex was extracted using AllPrep DNA/RNA/miRNA Universal Kit (Qiagen). Five microgram mouse genomic DNA was spiked with 0.02% unmethylated cl857 Sam7 Lambda DNA (Promega) and sonicated into 200 bp fragments with Covaris M2 (Covaris). After purification (PureLink PCR Purification Kit, Invitrogen), DNA fragments were then subjected to end repair with the end repair enzyme mix (NEB), dA tailing using Klenow 3′-5′ exo- (NEB) with purification at each step. Ligation with cytosine-methylated Illumina TruSeq DNA adapters were performed at 16 °C using T4 DNA ligase (NEB) overnight. The adapter-ligated DNA was then digested with MseI and MluCI (NEB) at 37 °C for 1 h. After purification, DNA fragments were subject to bisulfite conversion using the EpiTect Bisulfite Kit (Qiagen). After bisulfite conversion, the single-stranded uracil-containing DNA was subjected to 12 cycles of PCR reaction with Illumina TruSeq PCR primers and 2.5 U Pfu TurboCx Hotstart DNA polymerase (Agilent) to recover enough DNA for sequencing on Hiseq 4000 platform with 75 bp paired end mode (Illumina).

Sequencing bases with low quality in reads were trimmed by a customized Perl script. Adapters in reads were removed by Cutadapt. After trimming, sequencing reads were mapped to mm10 using Bismark with Bowtie2 and duplication reads were removed by a customized Perl script. Fisher Exact test was used to evaluate the significance of differential methylation on CpG site^17^. In order to control FDR, a sequential permutation method is employed^56^. A total of 1,000 permutations were performed for each CpG site. The number of true null hypotheses (m0) was estimated by a histogram method^17^. Based on the estimated m0, the adjusted p-value for each CpG site was calculated. DMSs were identified with adjusted p-value lower than or equal to 0.05. To determine DMRs, we developed a two-step approach. First, any two adjacent DMSs with at most 500 bp distance were merged into a cluster. In each of clusters which include at least 5 CpG sites, at least 80% of DMSs are prone to be methylated or unmethylated in one of the conditions. All clusters filled requirements above will be considered as DMR candidates. Second, at least 80% of CpG sites in a candidate DMR are prone to be methylated or unmethylated in one of conditions and each CpG site was required to have at least 0.1 methylation differences.

### RNA-seq library construction and data analysis

Total RNA from mouse frontal cortex was extracted using AllPrep DNA/RNA/miRNA Universal Kit (Qiagen). RNA-seq libraries were constructed using the TruSeq Stranded mRNA Library Preparation Kit (Illumina) following the manufacturer’s instructions. Briefly, the polyA-containing mRNA molecules were enriched from 500 ng total RNA via two rolls of oligo-dT magnetic beads purification. The resultant mRNA was fragmented and primed into first strand cDNA using reverse transcriptase and random primers, followed by the removal of the RNA template and synthesis of the second strand to generate blunt-ended ds cDNA. Then a single ‘A’ nucleotide was added to the 3′ ends of the blunt fragments and indexing adapter was ligated to the ends of the ds cDNA. Those DNA fragments with adapter molecules on both ends were enriched by PCR amplification for 12 cycles. After Ampure XP beads purification, the PCR product was size-selected with the range from 220 to 500 bp on 2% dye-free agarose gel using pippin recovery system (Sage Science). The recovered libraries were sequenced on Hiseq 4000 platform with 75 bp paired end mode (Illumina). After trimming bases of low quality and removing adapters, reads were mapped to mm10 by RSEM^57^ with Bowtie2. The raw counts were employed to identify differentially expression genes by DESeq2^58^. The definition of differentially expression genes includes two requirements: (1) the *p*-value adjusted is less than 0.05, and (2) there are at least 1.5 fold changes. The visualized data normalized to 1 million was generated by Bedtools^59^.

### GO analysis

GO analysis was performed via the Gene Functional Annotation Tool at the DAVID^60^ website (https://david.ncifcrf.gov/, version 6.8). Default parameters were used for the enrichment analysis for BP, cellular component (CC), and molecular function (MF). The resulting GO terms and the corresponding *p*-values were then processed using REVIGO^61^ to remove redundancy. The ten most significant BP categories were shown.

### Reporting summary

Further information on research design is available in the Nature Research Reporting Summary linked to this article.

## Supplementary information

Supplementary Information

Reporting summary

Description of Additional Supplementary Files

Supplementary Data 1

Supplementary Data 2

Supplementary Data 3

Supplementary Data 4

Supplementary Data 5

Supplementary Data 6

Supplementary Data 7

Supplementary Data 8

Source Data

## Data Availability

The datasets generated during and/or analysed during the current study are available in the NCBI Gene Expression Ominibus (GEO), GSE108768 (including GSE108750, GSE108762, and GSE124671). Publicly available brain “omics” data used in this manuscript are summarized in Supplementary Data 8. The full and original western blots used for Fig. 2 and Supplementary Fig. 9 are provided in Source Data file.
