# Supplementary material for: EGR1 recruits TET1 to shape the brain methylome during development and upon neuronal activity
Source: Nat Commun. 2019 Aug 29;10:3892. doi: 10.1038/s41467-019-11905-3 (PMC6715719; doi:10.1038/s41467-019-11905-3)
Supplement: Supplementary file 1 — Supplementary Information [file 41467_2019_11905_MOESM1_ESM.docx]

**Supplementary Information**

**EGR1 Recruits TET1 to Shape the Brain Methylome during Development and upon Neuronal Activity**

**Contents:**

Supplementary Figures 1-23


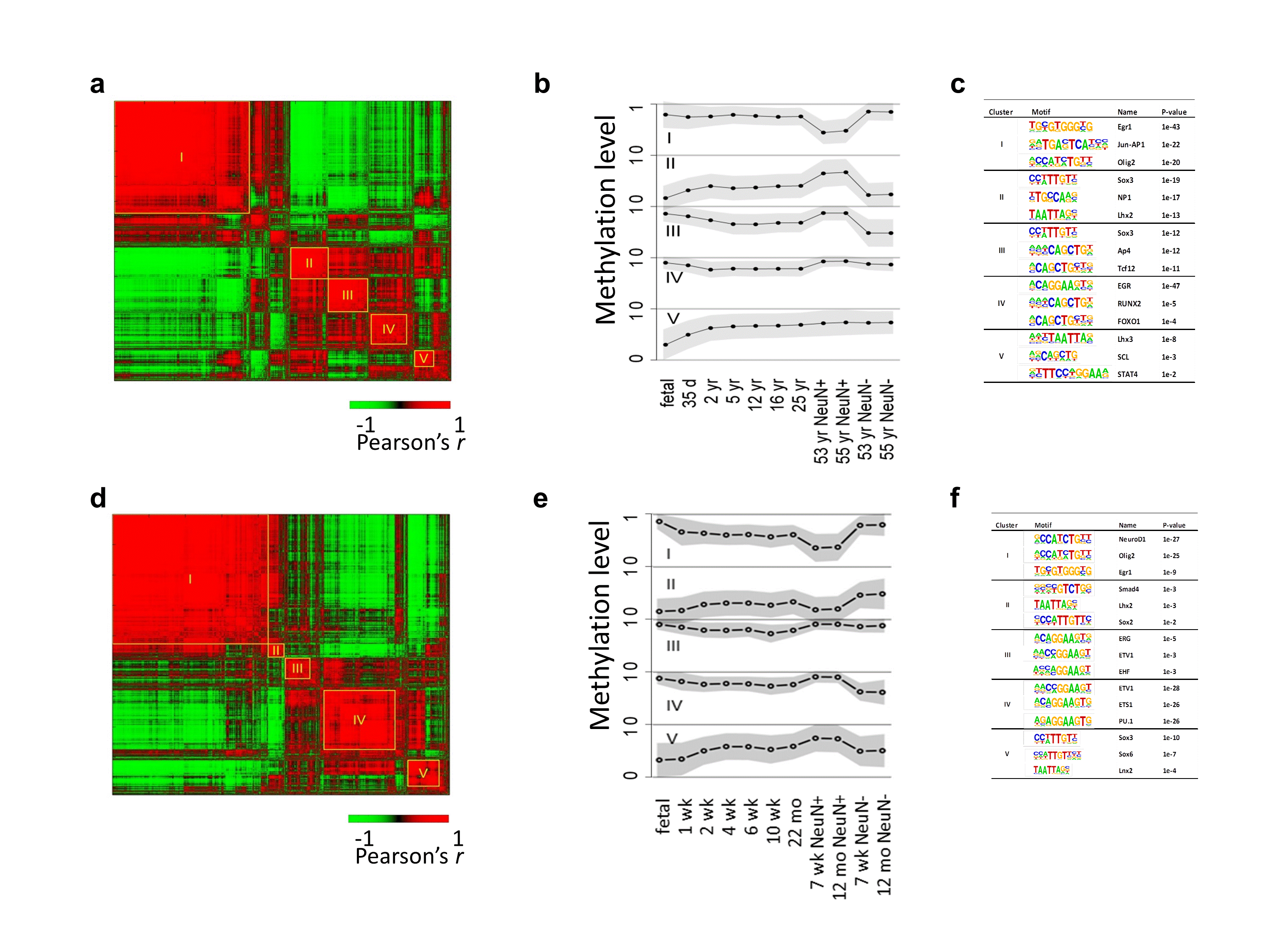


**Supplementary** **Figure 1. Co-methylation analysis of bipolar methylated loci during mouse and human brain development.** (a, d) Heatmaps displaying the hierarchical clustering of human and mouse bipolar methylated loci, respectively. The top five largest co-methylated modules are marked with yellow squares. (b, e) The methylation level distributions of five co-methylated modules for human and mouse, respectively. The black solid line shows the average, and the grey color shows the standard deviation. (c, f) Top TF motifs enriched in each module identified in human and mouse brain methylomes, respectively (for the full lists, see **Supplementary Table 1**).

**
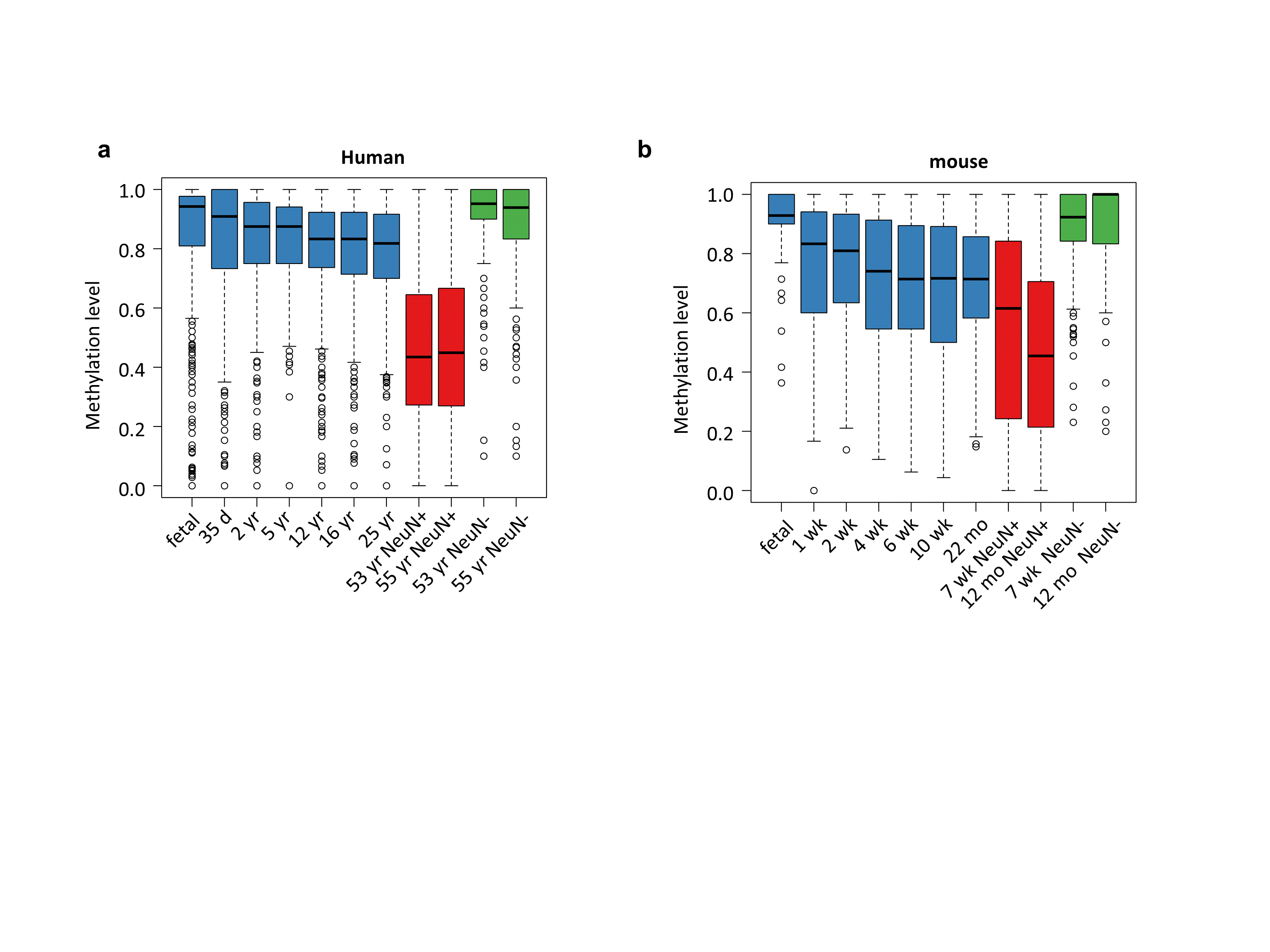
**

**Supplementary Figure 2. Methylation profiles of EGR1 binding sites predicted within co-methylation module I.** The methylation profiles of the predicted EGR1 binding sites during human (a) and mouse (b) brain development and cell specifications. Only binding sites with at least ten methylation calls in every methylome were included.

**
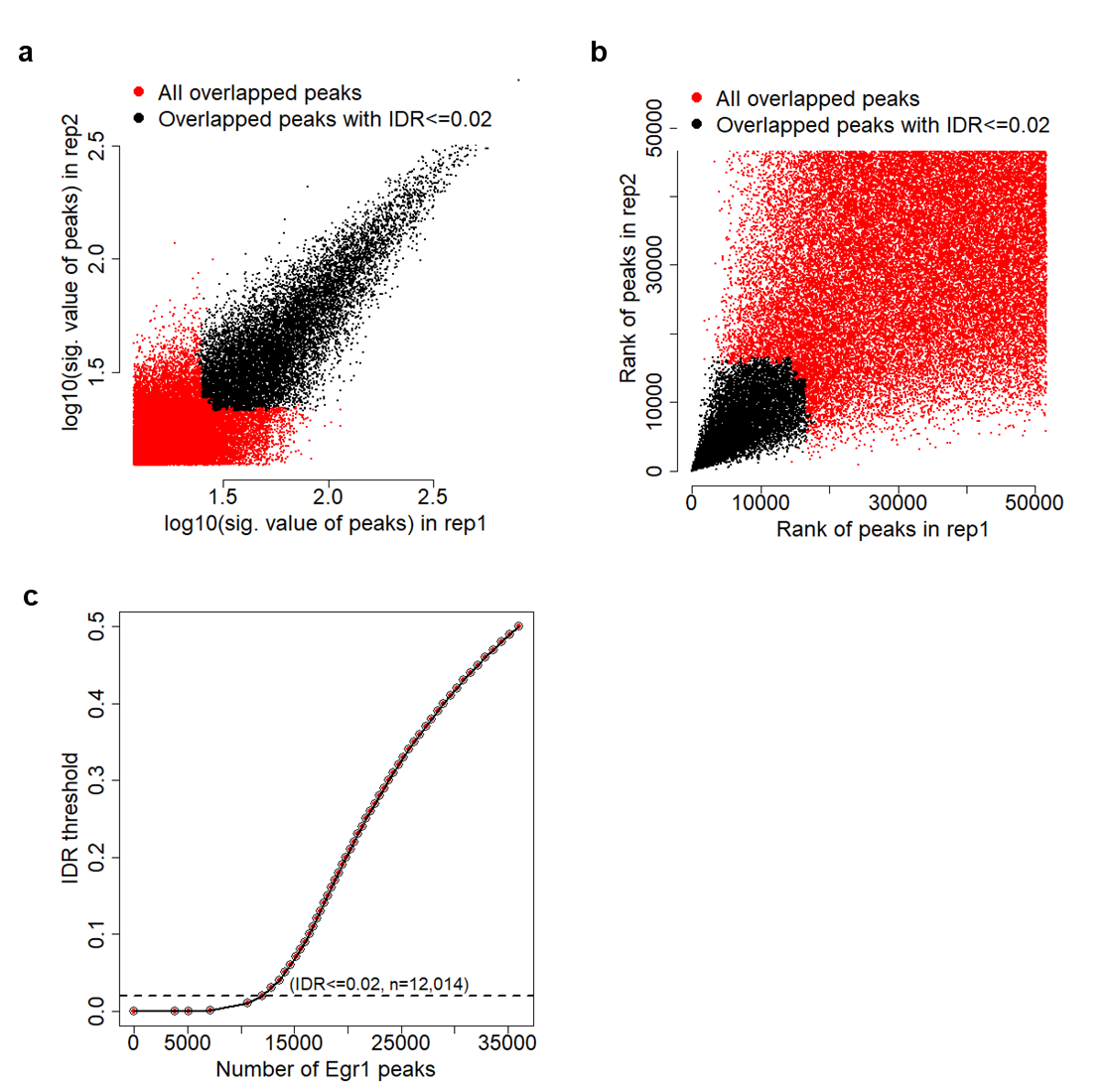
**

**Supplementary Figure 3. Consistency of two biological replicates for EGR1 ChIP-Seq assays.** (a) Scatter plots of signal scores for peaks that overlap in two biological replicates. IDR: irreproducible discovery rate. (b) Scatter plots of ranks for peaks that overlap in two biological replicates. Note that low ranks correspond to high signals, and vice versa. (c) The estimated IDR as a function of different rank thresholds. (a, b) Black data points represent peak pairs that pass an IDR threshold of 2%, whereas the red data points represent peak pairs that do not pass the IDR threshold of 2%. Two EGR1 ChIP-seq replicates show high reproducibility with 12,014 peaks passing 2% IDR threshold.

**
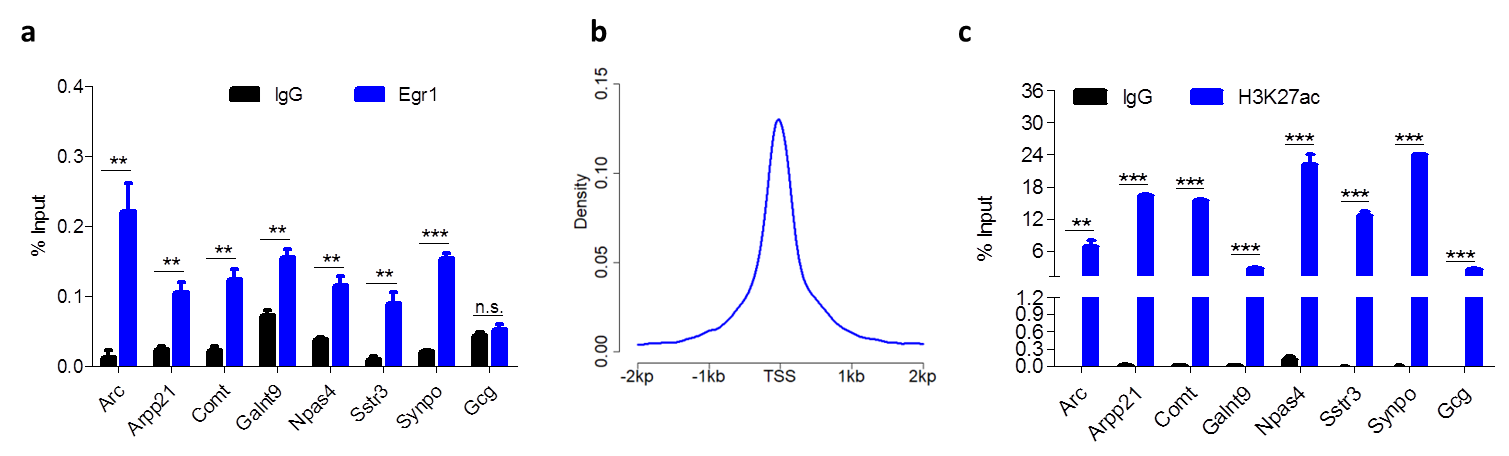
**

**Supplementary Figure 4. Enrichment of EGR1 peaks around TSS and ChIP-qPCR validation for EGR1 binding and histone mark H3K27ac.** (a, c) ChIP-qPCR analysis for EGR1 (a) and H3K27ac (c) at eight genomic regions. *Arc* and *Gcg* are positive and negative controls for EGR1 binding, respectively. (b) Enrichment of EGR1 peaks around TSS. Analysis is by t-test, *, p<0.05, **, p<0.01, ***, p<0.001. n.s., not significant. Error bars ± standard deviation (s.d.) from three technical replicates.

**
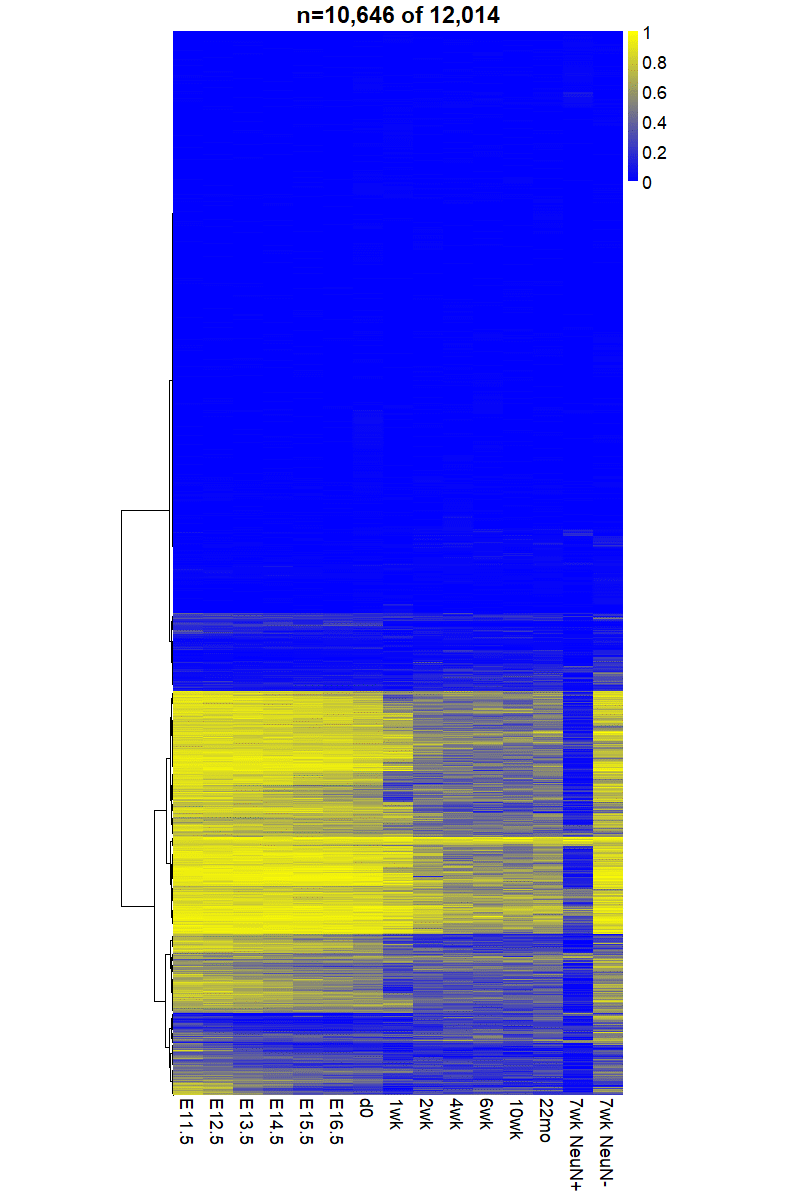
**

**Supplementary Figure 5. Methylation profiles of EGR1 peaks during mouse brain development.** Methylation profiles of EGR1 peaks in mouse frontal cortices from embryonic day 11.5 to 22 months, neuron and glia at 7 weeks. The average of methylation levels within EGR1 binding sites were calculated based on CpG sites with at least 5 reads covered. EGR1 binding sites with missing data in any of the 15 samples were discarded.

**
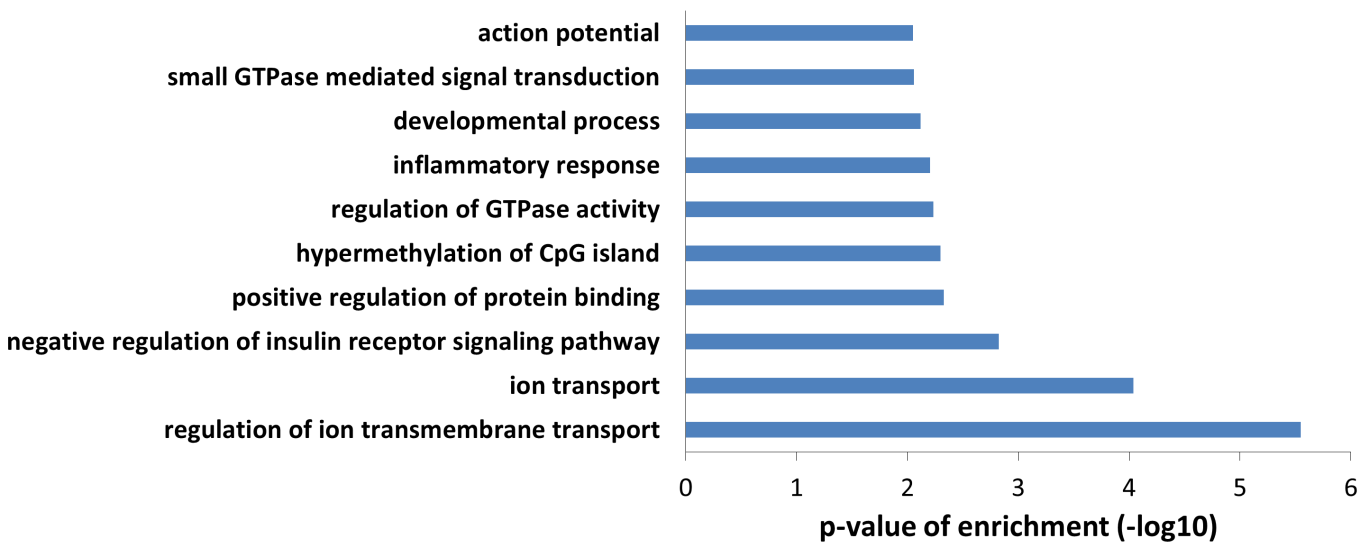
**

**Supplementary Figure 6. GO analysis for genes (n = 598, which have been annotated by DAVID) with TSS flanking 10 kb of EGR1 peaks.** Genes were identified with 1,925 EGR1 peaks which show decreased methylation during frontal cortex development and hypomethylated in adult neurons compared to that in adult glial cells

**
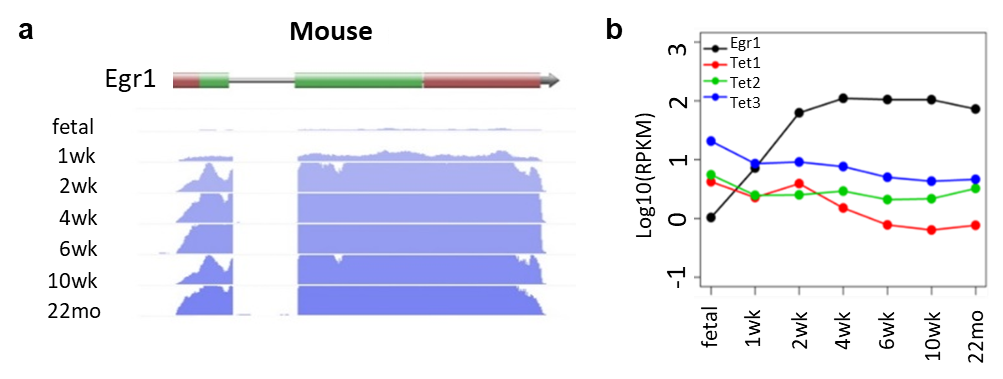
**

**Supplementary Figure 7. Expression profiles of *Egr1*, *Tet* genes during mouse brain development.** (a) Transcript abundance for *Egr1* and (b) three *Tet* genes based on RNA-Seq data for frontal cortices during mouse brain development. RPKM, reads per kilobase of exon per million fragments mapped.

**
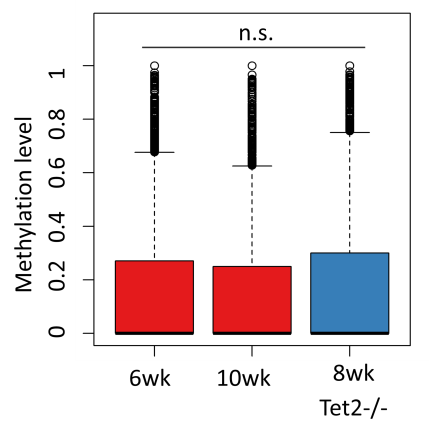
**

**Supplementary Figure 8. Methylation profiles of EGR1 binding sites in frontal cortices of wild type mice at 6-week, 10-week, and Tet2 knockout (Tet2-/-) mice at 8-week.** n.s., not significant.


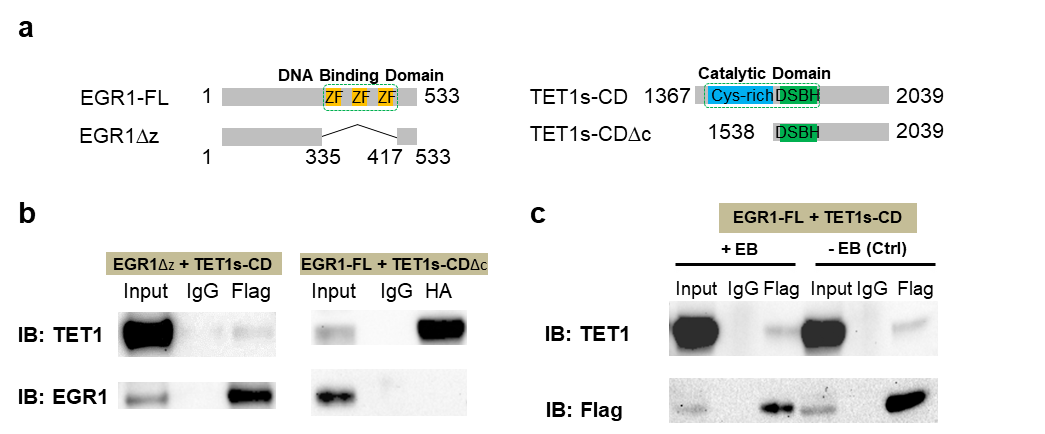


**Supplementary Figure 9. Interaction between EGR1 and TET proteins by co-immunoprecipitation.** (a) Diagrams show EGR1-FL (533 aa), EGR1∆z (residues 1-335, 417-533) with 81 aa removed to delete the zinc finger domain, TET1s-CD (residues 1367-2039) and TET1s-CD∆c(residues 1538-2039) with 171 aa removed to delete the cystine-rich domain. (b) Co-immunoprecipitation of EGR1∆z and TET1s-CD (right) and EGR1-FL and TET1s-CD∆c. (c) Co-immunoprecipitation of EGR1-FL and TET1s-CD with (right) and without (left) the presence of ethidium bromide (EB). Source data are provided as a Source Data file.

**
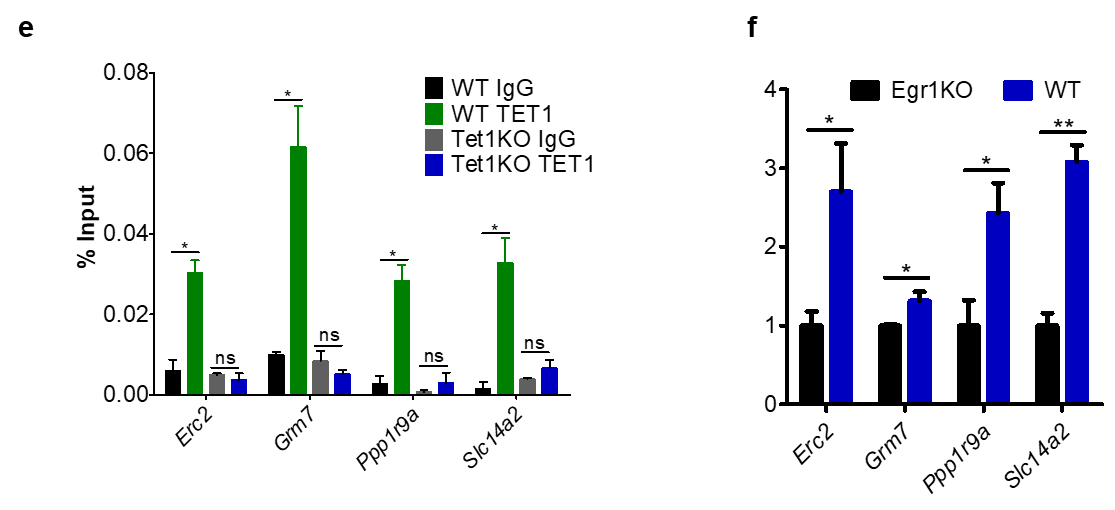
**

**Supplementary Figure 10. The overlapping rate of TET1 ChIP-seq libraries and the genome distribution of TET1 ChIP-seq peaks.** (a&b) The overlapping statuses of TET1 ChIP-seq peaks between two biological replicates generated with antibodies 5D6 and 91171 (Active Motif) for mouse frontal cortices from Egr1KO (left) and WT (right) mice. (c&d) The genome distribution of TET1 ChIP-seq peaks identified for mouse frontal cortices from Egr1KO (left) and WT (right) mice. (e) TET1 ChIP-qPCR in the frontal cortices of Tet1WT and Tet1KO mice. (f) TET1 ChIP-qPCR in the frontal cortices of Egr1WT and Egr1KO mice. TET1 enrichment in the frontal cortices of Egr1WT is normalized to the enrichment in Egr1KO mice. ChIP-seq TET1 antibody 5D6 (61741, Active Motif) was used for figures e and f. Analysis is by t-test, *, p<0.05, **, p<0.01, ***, p<0.001. ns, not significant. Error bars ± standard deviation (s.d.) from three technical replicates.


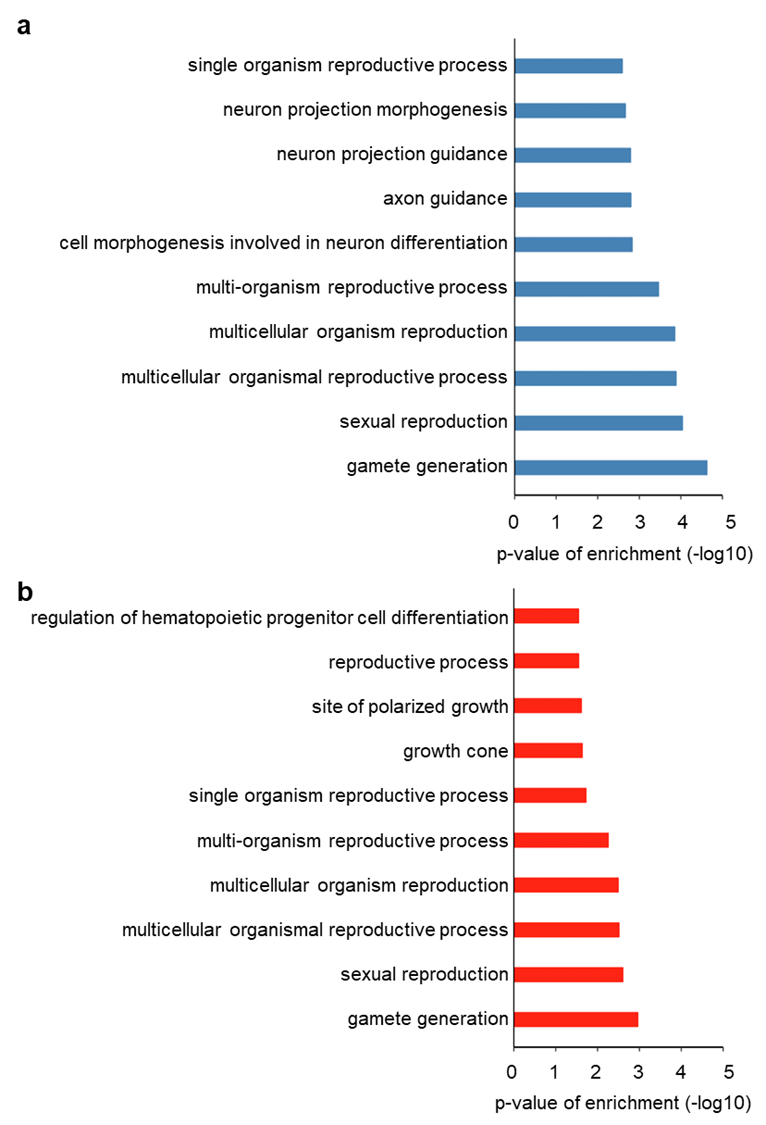


**Supplementary Figure 11. GO annotation of genes associated with TET1 ChIP peaks identified for mouse frontal cortices from WT (a) and Egr1KO (b) mice.**

**Supplementary Figure 12. Characterizations of primary culture of mouse cortical neurons isolated at E16.5.** E16.5 mouse cortical neurons were cultured *in vitro* for 7 days and co-immunostained with Tuj-1 and GFAP. Images from two view fields were shown (scale bar=200µm).


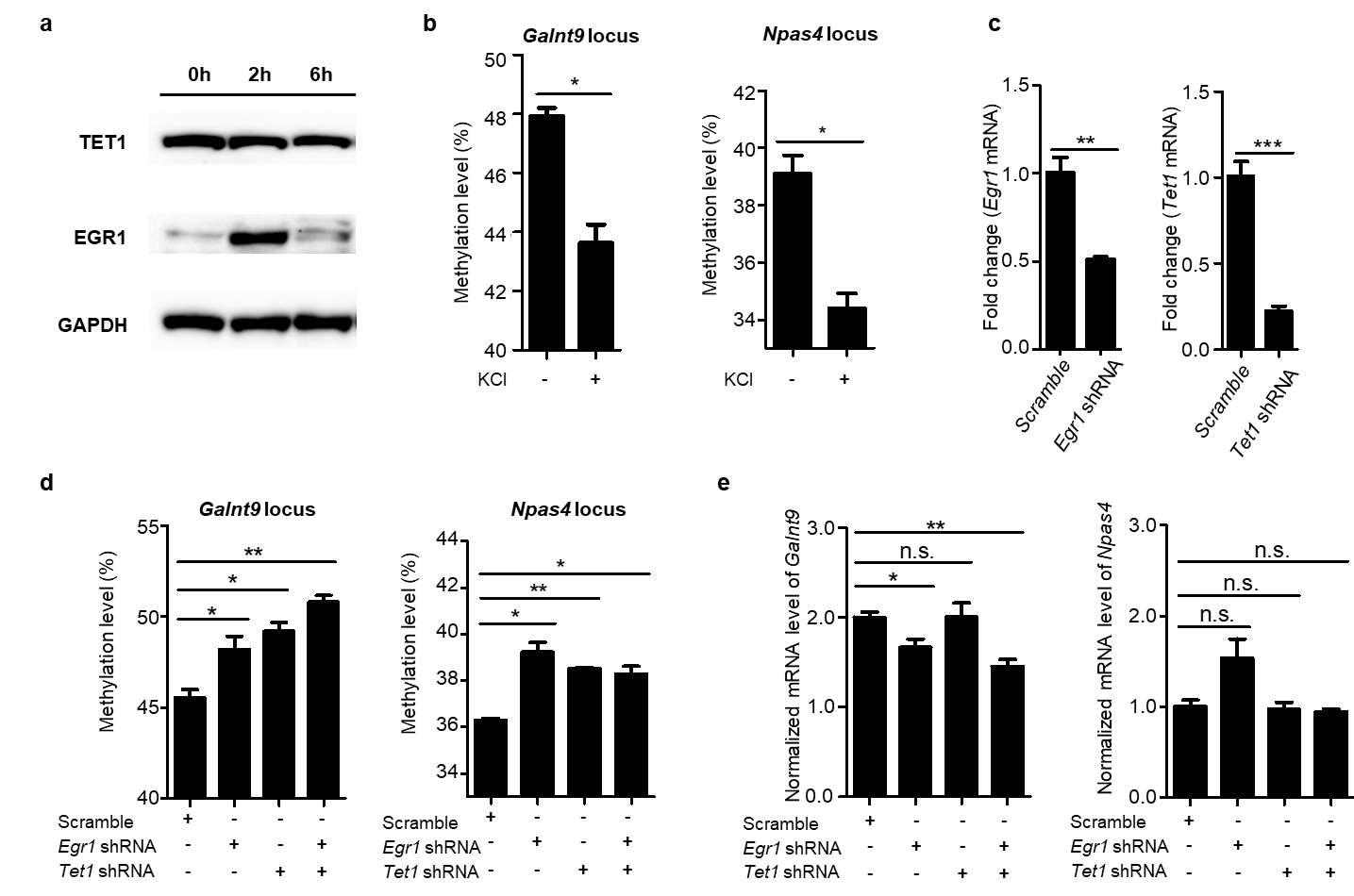


**Supplementary Figure 13. Epigenetic regulation of EGR1 and TET1 on its target loci.** (a) The time-course expression of EGR1 and TET1 proteins in E16.5 cortical neurons upon KCl stimulation. GAPDH serves as a loading control. (b) Methylation changes of *Galnt9* and *Npas4* loci upon KCl stimulation. (c) Knockdown of *Egr1* and *Tet1* in E16.5 cortical neurons via lentivirus-mediated delivery of shRNA. (d) Methylation level of Galnt9 and Npas4 loci determined by pyrosequencing and (e) expression level of *Galnt9* and *Npas4* mRNA determined by qRT-PCR in E16.5 cortical neurons under different knockdown conditions. P-values were determined by t-test, *, P<0.05, **, P<0.01. ***, p<0.001. Values represent mean ± SD from three biological replicates.

**
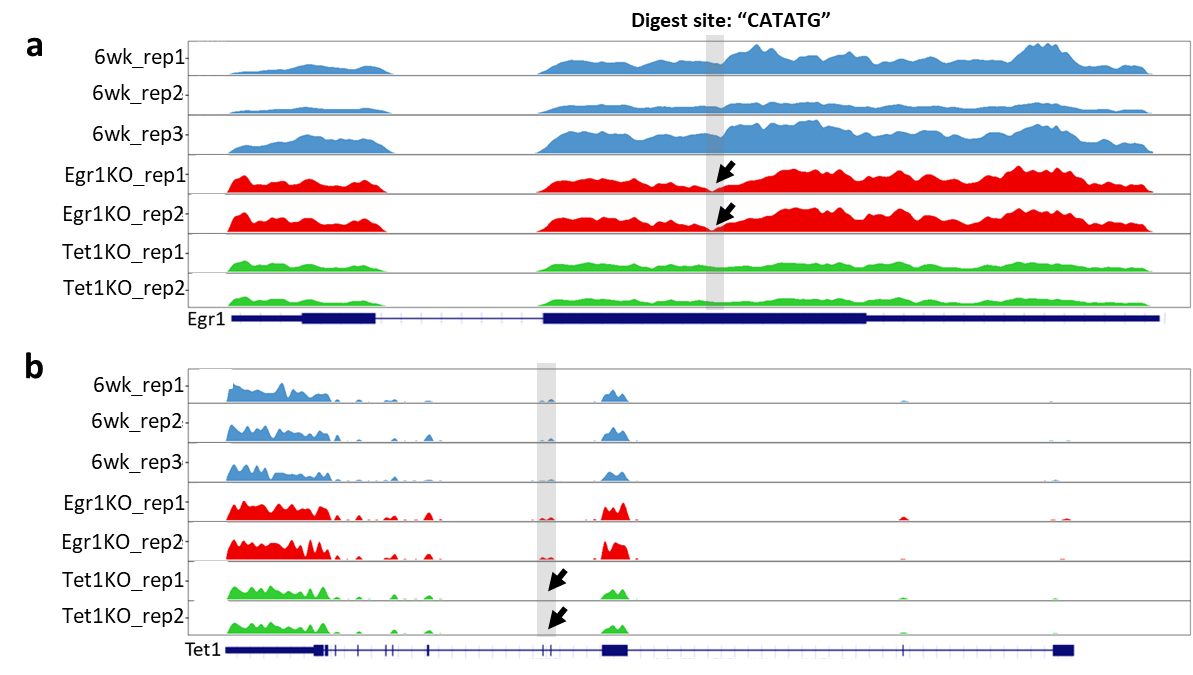
**

**Supplementary Figure 14. Visualization of read coverage along (a) *Egr1* and (b) *Tet1* genes.** Gray boxes highlight the genomic regions and black arrows indicate specific genetic aberrations in the genomes of Egr1KO and Tet1KO mice.

**
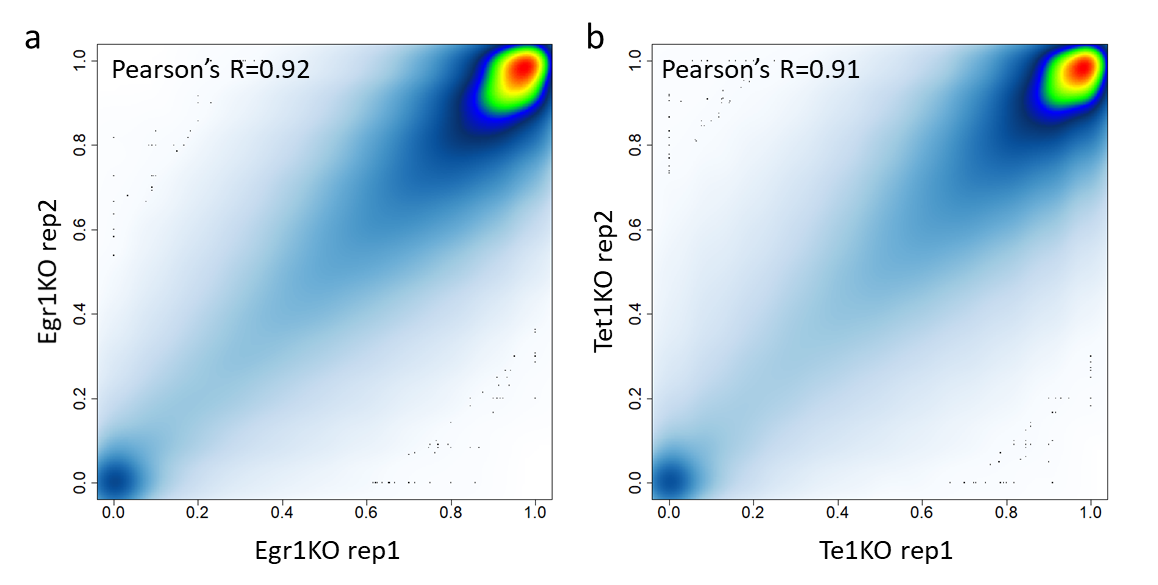
**

**Supplementary Figure 15. Consistency of methylation on pairwise replicate.** Consistency of RRBS-seq for two biological replicates of (a) Egr1KO mice and (b) Tet1KO mice. Methylation levels were calculated with CpG sites with at least 10 reads covered. Colors represent the density of CpG sites, in which red color denotes high density and blue color denotes low density. Black points represent the areas of lowest regional density.

**
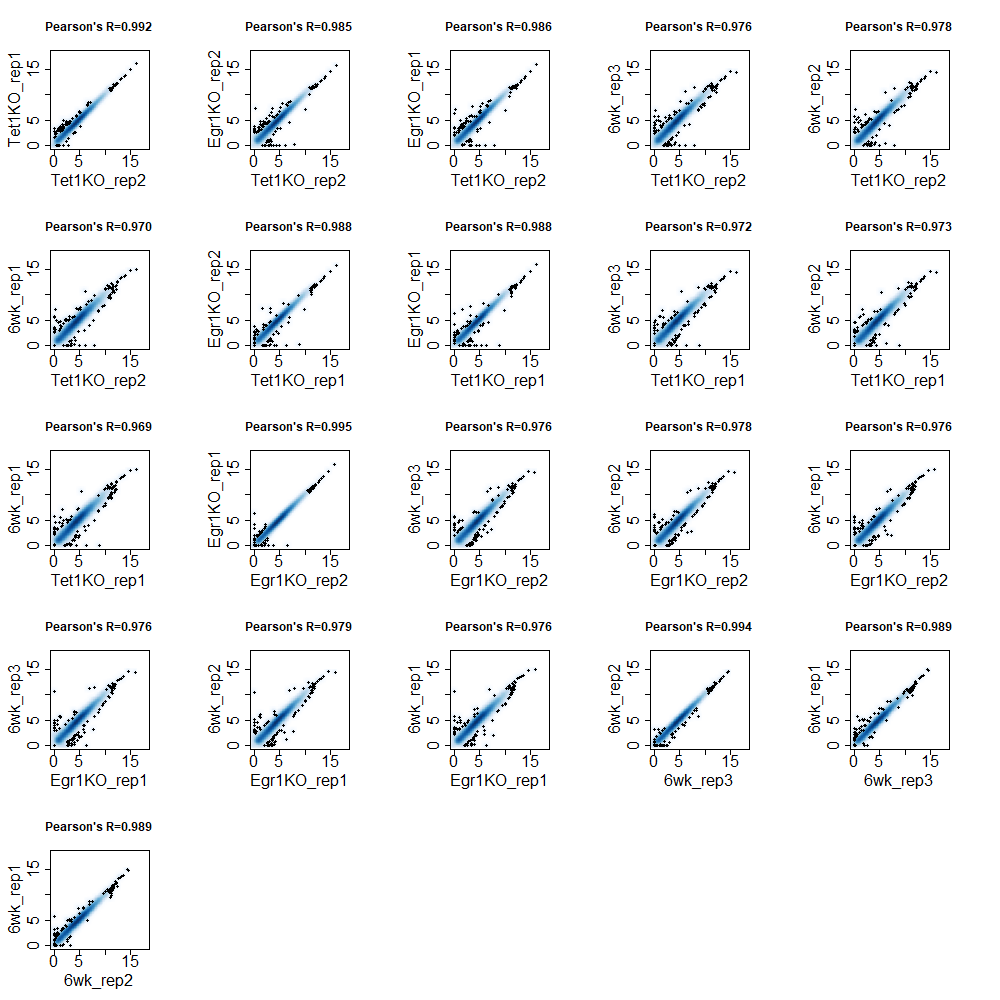
**

**Supplementary Figure 16. Correlation analysis of the transcriptomes derived from the frontal cortices of Egr1KO, Tet1KO and wild type mice.** Scatter plots represent the correlations of any sample pair. Each black point denotes a gene. Dark blue denotes high density of points. The expression level for each gene was presented by log2TPM. Two biological replicates were included in the analyses for Egr1KO or Tet1KO mice and three biological replicates were included for age/gender-matched wild type mice.

**
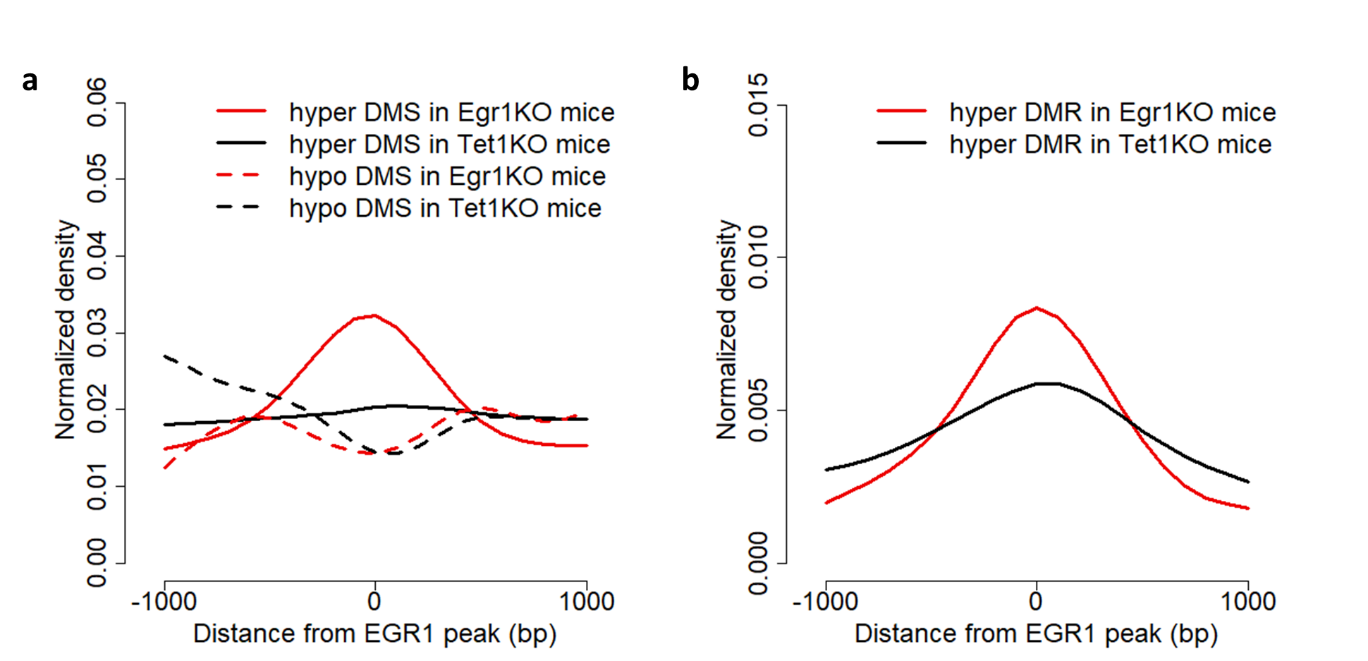
**

**Supplementary Figure 17. Distribution of (a) differentially methylated CpG sites (DMSs) and (b) differentially methylated regions (DMRs) surrounding EGR1 binding sites.** Red color and black color represent differentially methylated loci from Egr1KO mice and Tet1KO mice, respectively. Solid line and dash line denote hypermethylated loci and hypomethylated loci, respectively.

**
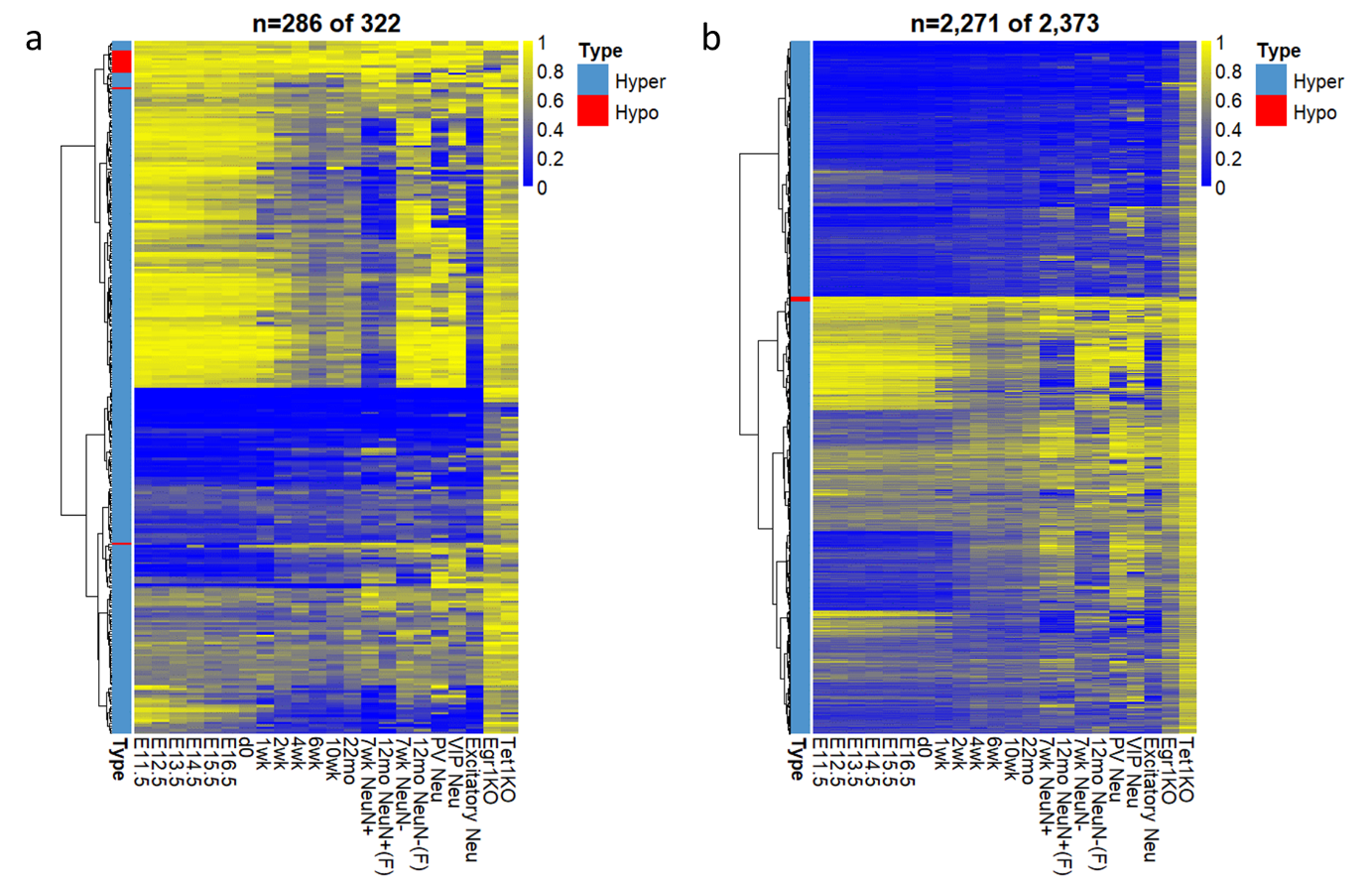
**

**Supplementary Figure 18. Methylation profiles of DMRs identified from (a) Egr1KO mice and in (b) Tet1KO mice during mouse brain development and cell types.** The average of methylation levels within DMRs were calculated with CpG sites with at least 5 reads covered. Blue and red color bars represent hypermethylated and hypomethylated DMRs, respectively.

**
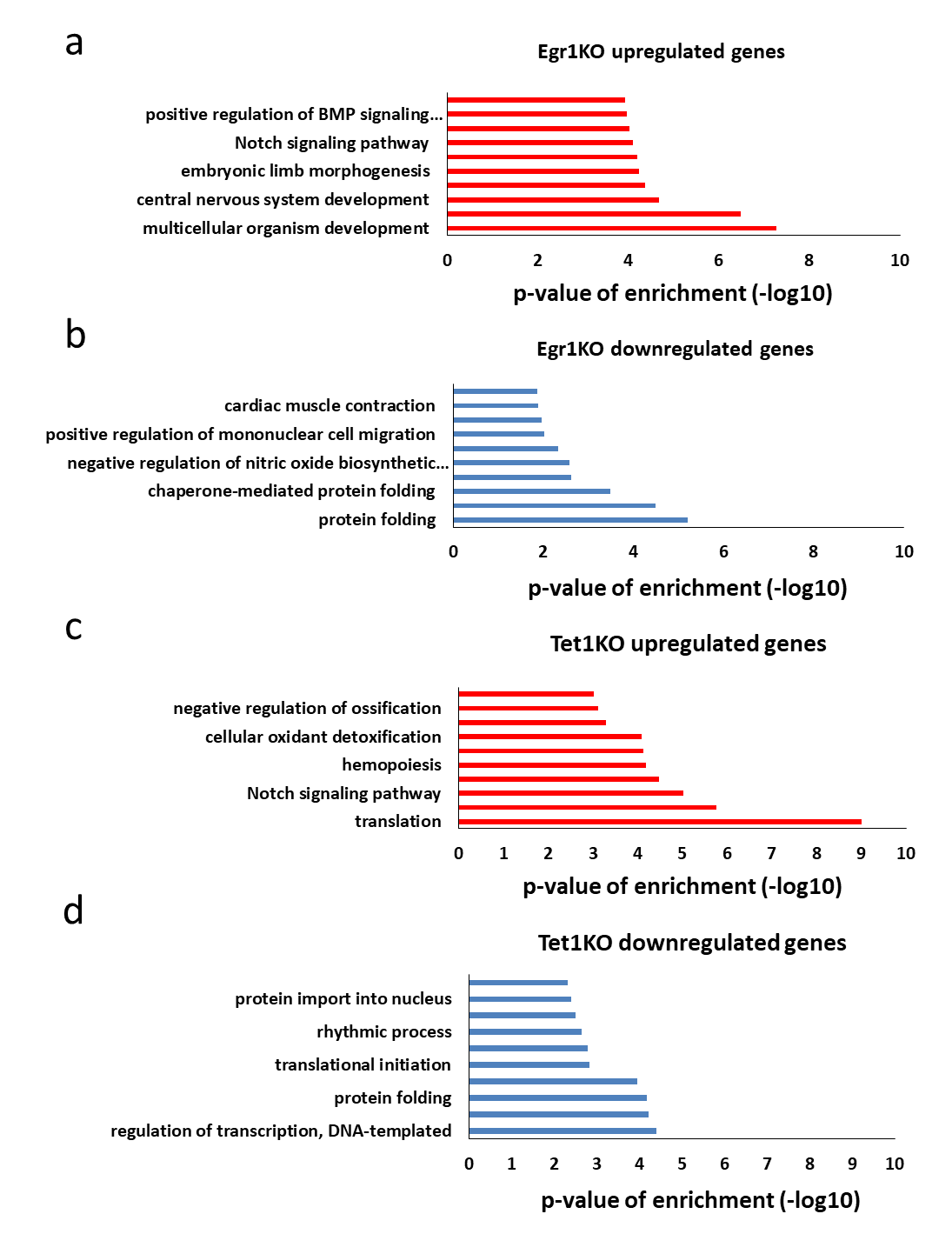
**

**Supplementary Figure 19. GO analysis of differentially expressed genes identified in Egr1KO or Tet1KO frontal cortices.** GO analysis of **(**a) upregulated genes in Egr1KO, (b) downregulated genes in Egr1KO, (c) upregulated genes in Tet1KO and (d) downregulated genes in Tet1KO.

**
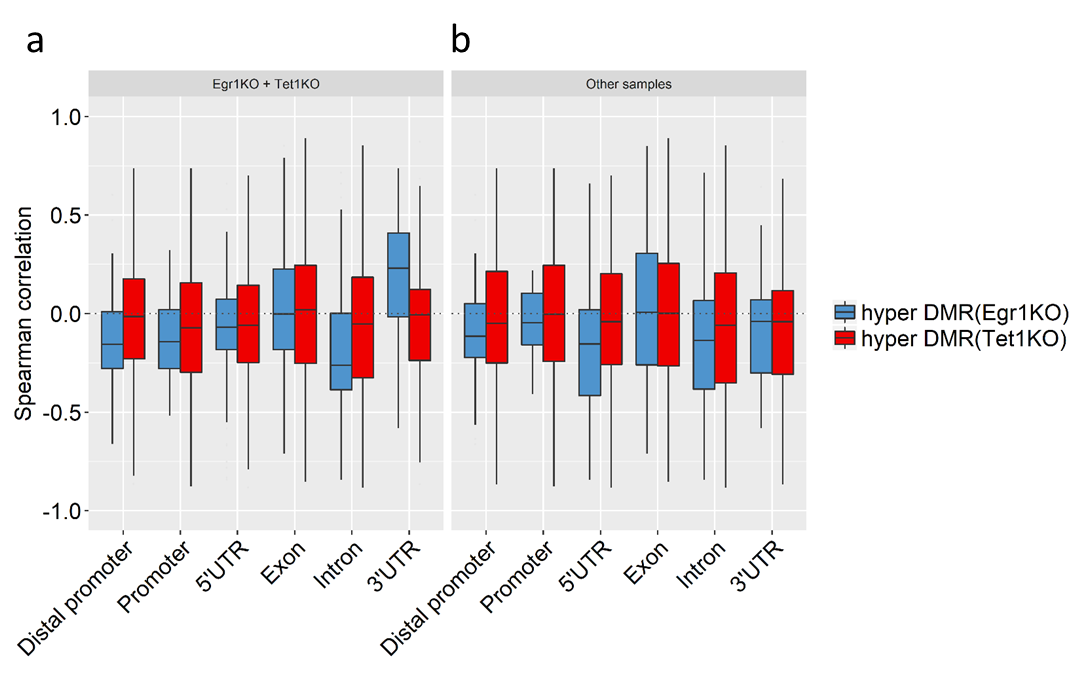
**

**Supplementary Figure 20. Spearman’s correlation between DMR methylation and adjacent gene expression.** (a) Correlations were calculated across genes overlapped with DMRs in Egr1KO mice and Tet1KO mice. (b) Correlations were calculated for genes overlapped with DMRs across 33 mouse brain samples with methylome and transcriptome data available (Supplementary Table 4). Blue and red color bars represent hypermethylated DMRs from Egr1KO mice and Tet1KO mice relative to 6 week old wild type mice, respectively.

**
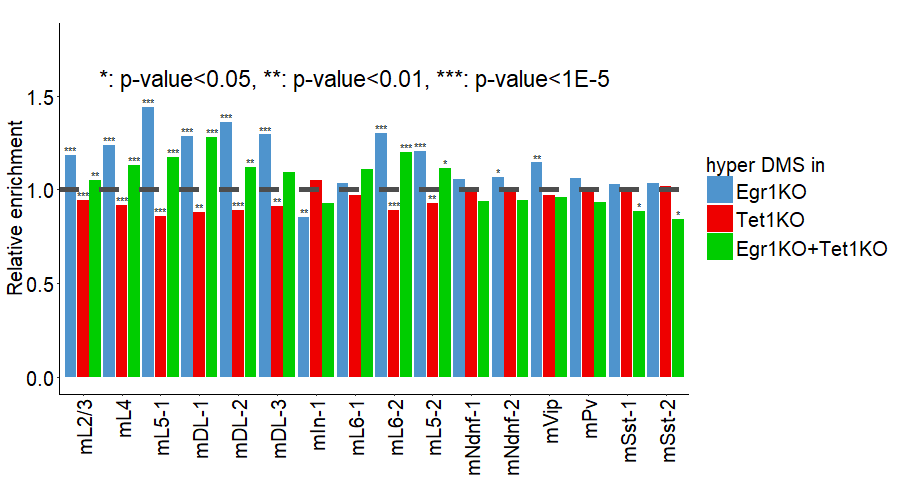
**

**Supplementary Figure 21. Relative enrichment of hypermethylated DMSs on neuron subtype specific DMRs.** Blue, red and green color represent the hypermethylated DMS from Egr1KO mice only, Tet1KO mice only, in both Egr1KO mice and Tet1KO mice, respectively. The neuron subtype specific DMRs were reported by Luo *et al* (Science, 2017). The enrichment significance was evaluated using Fisher Exact test across 16 neuron subtypes. Enrichment score denotes odds ratio and all hypermethylated DMSs were used as controls for three individual lists.

**
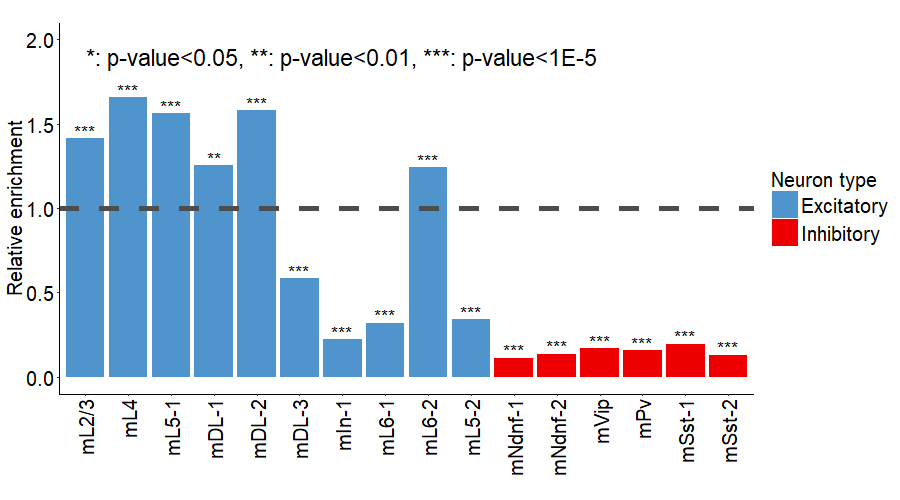
**

**Supplementary Figure 22. Relative enrichment of EGR1 binding sites on neuron subtype specific DMRs.** Blue color represents excitatory neuron subtypes and red color denotes inhibitory neuron subtypes. The significance of enrichment was evaluated by Fisher Exact test across 16 neuron subtypes. Enrichment score denote odds ratio and all 16 neuron subtypes together were used as controls for each individual neuron subtype.

**
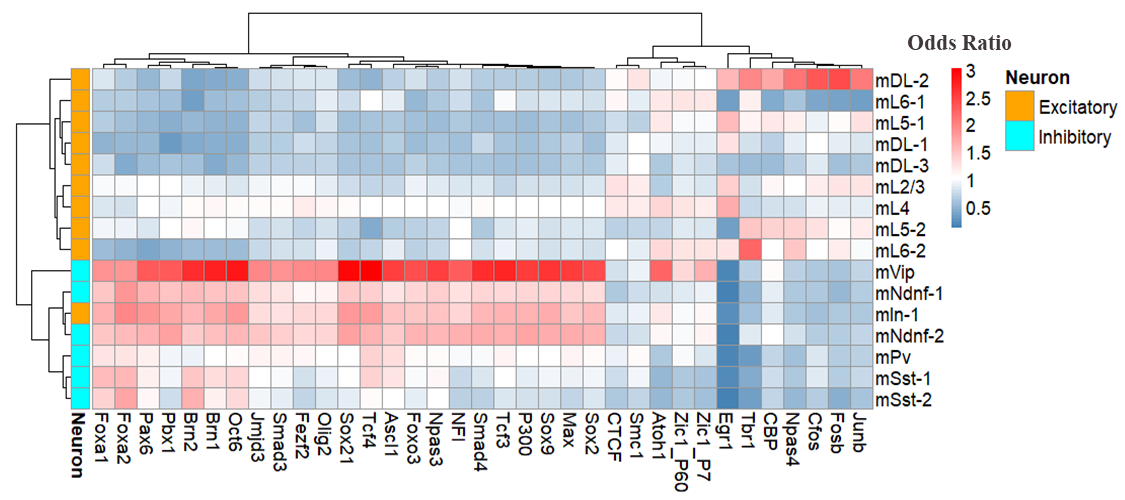
**

**Supplementary Figure 23. Clustering of TFs relatively enriched on neuron subtype specific DMRs.** Orange color in heatmap represents excitatory neuron subtypes and cyan color denotes inhibitory neuron subtypes. The significance of enrichment was evaluated by Fisher Exact test across 16 neuron subtypes. Enrichment score in each entity of matrix denote odds ratio and all 16 neuron subtypes together were used as controls for each individual neuron subtype.
