# Supplementary material for: EGR1 recruits TET1 to shape the brain methylome during development and upon neuronal activity
Source: Nat Commun. 2019 Aug 29;10:3892. doi: 10.1038/s41467-019-11905-3 (PMC6715719; doi:10.1038/s41467-019-11905-3)
Supplement: Supplementary file 2 — Reporting summary [file 41467_2019_11905_MOESM2_ESM.pdf]

nature research

Corresponding author(s): Hehuang Xie

Last updated by author(s): Aug 2, 2019

Reporting Summary

Nature Research wishes to improve the reproducibility of the work that we publish. This form provides structure for consistency and transparency in reporting. For further information on Nature Research policies, see Authors & Referees and the Editorial Policy Checklist .

Statistics

For all statistical analyses, confirm that the following items are present in the figure legend, table legend, main text, or Methods section.

n/a	Confirmed	
☐	☒	The exact sample size (*n*) for each experimental group/condition, given as a discrete number and unit of measurement
☐	☒	A statement on whether measurements were taken from distinct samples or whether the same sample was measured repeatedly
☐	☒	The statistical test(s) used AND whether they are one- or two-sided<br>*Only common tests should be described solely by name; describe more complex techniques in the Methods section.*
☒	☐	A description of all covariates tested
☒	☐	A description of any assumptions or corrections, such as tests of normality and adjustment for multiple comparisons
☐	☒	A full description of the statistical parameters including central tendency (e.g. means) or other basic estimates (e.g. regression coefficient) AND variation (e.g. standard deviation) or associated estimates of uncertainty (e.g. confidence intervals)
☐	☒	For null hypothesis testing, the test statistic (e.g. *F*, *t*, *r*) with confidence intervals, effect sizes, degrees of freedom and *P* value noted<br>*Give P values as exact values whenever suitable.*
☒	☐	For Bayesian analysis, information on the choice of priors and Markov chain Monte Carlo settings
☒	☐	For hierarchical and complex designs, identification of the appropriate level for tests and full reporting of outcomes
☒	☐	Estimates of effect sizes (e.g. Cohen's *d*, Pearson's *r*), indicating how they were calculated

Our web collection on statistics for biologists contains articles on many of the points above.

Software and code

Policy information about availability of computer code

Data collection	All computer code and software used to collect the data were described in details in the Methods section.
Data analysis	All computer code and software used to collect the data were described in details in the Methods section.

For manuscripts utilizing custom algorithms or software that are central to the research but not yet described in published literature, software must be made available to editors/reviewers. We strongly encourage code deposition in a community repository (e.g. GitHub). See the Nature Research guidelines for submitting code & software for further information.

Data

Policy information about availability of data

All manuscripts must include a data availability statement. This statement should provide the following information, where applicable:

- Accession codes, unique identifiers, or web links for publicly available datasets
- A list of figures that have associated raw data
- A description of any restrictions on data availability

The datasets generated during and/or analysed during the current study are available in the NCBI Gene Expression Ominibus (GEO), GSE108750, GSE108762, GSE108768, and GSE124671. Publicly available brain “omics” data used in this manuscript are summarized in Supplementary Data 8. The full and original western blots used for Figure 2 and Supplementary Figure 9 are provided in Supplementary Data 9.

Field-specific reporting

Please select the one below that is the best fit for your research. If you are not sure, read the appropriate sections before making your selection.

☒ Life sciences ☐ Behavioural & social sciences ☐ Ecological, evolutionary & environmental sciences

For a reference copy of the document with all sections, see nature.com/documents/nr-reporting-summary-flat.pdf

Life sciences study design

All studies must disclose on these points even when the disclosure is negative.

Sample size	Sample sizes were chosen based on material available ensuring that it will be appropriate for statistical analysis.
Data exclusions	No data exclusions
Replication	All ChIP-seq, RRBS, RNA-seq data includes two biological replicates. For luciferase reporter assays and loci-specific methylation assay, three biological replicates are included.
Randomization	n/a
Blinding	n/a

Behavioural & social sciences study design

All studies must disclose on these points even when the disclosure is negative.

Study description	*Briefly describe the study type including whether data are quantitative, qualitative, or mixed-methods (e.g. qualitative cross-sectional, quantitative experimental, mixed-methods case study).*
Research sample	*State the research sample (e.g. Harvard university undergraduates, villagers in rural India) and provide relevant demographic information (e.g. age, sex) and indicate whether the sample is representative. Provide a rationale for the study sample chosen. For studies involving existing datasets, please describe the dataset and source.*
Sampling strategy	*Describe the sampling procedure (e.g. random, snowball, stratified, convenience). Describe the statistical methods that were used to predetermine sample size OR if no sample-size calculation was performed, describe how sample sizes were chosen and provide a rationale for why these sample sizes are sufficient. For qualitative data, please indicate whether data saturation was considered, and what criteria were used to decide that no further sampling was needed.*
Data collection	*Provide details about the data collection procedure, including the instruments or devices used to record the data (e.g. pen and paper, computer, eye tracker, video or audio equipment) whether anyone was present besides the participant(s) and the researcher, and whether the researcher was blind to experimental condition and/or the study hypothesis during data collection.*
Timing	*Indicate the start and stop dates of data collection. If there is a gap between collection periods, state the dates for each sample cohort.*
Data exclusions	*If no data were excluded from the analyses, state so OR if data were excluded, provide the exact number of exclusions and the rationale behind them, indicating whether exclusion criteria were pre-established.*
Non-participation	*State how many participants dropped out/declined participation and the reason(s) given OR provide response rate OR state that no participants dropped out/declined participation.*
Randomization	*If participants were not allocated into experimental groups, state so OR describe how participants were allocated to groups, and if allocation was not random, describe how covariates were controlled.*

Ecological, evolutionary & environmental sciences study design

All studies must disclose on these points even when the disclosure is negative.

Study description	*Briefly describe the study. For quantitative data include treatment factors and interactions, design structure (e.g. factorial, nested, hierarchical), nature and number of experimental units and replicates.*
Research sample	*Describe the research sample (e.g. a group of tagged Passer domesticus, all Stenocereus thurberi within Organ Pipe Cactus National Monument), and provide a rationale for the sample choice. When relevant, describe the organism taxa, source, sex, age range and any manipulations. State what population the sample is meant to represent when applicable. For studies involving existing datasets, describe the data and its source.*

Sampling strategy	*Note the sampling procedure. Describe the statistical methods that were used to predetermine sample size OR if no sample-size calculation was performed, describe how sample sizes were chosen and provide a rationale for why these sample sizes are sufficient.*
Data collection	*Describe the data collection procedure, including who recorded the data and how.*
Timing and spatial scale	*Indicate the start and stop dates of data collection, noting the frequency and periodicity of sampling and providing a rationale for these choices. If there is a gap between collection periods, state the dates for each sample cohort. Specify the spatial scale from which the data are taken*
Data exclusions	*If no data were excluded from the analyses, state so OR if data were excluded, describe the exclusions and the rationale behind them, indicating whether exclusion criteria were pre-established.*
Reproducibility	*Describe the measures taken to verify the reproducibility of experimental findings. For each experiment, note whether any attempts to repeat the experiment failed OR state that all attempts to repeat the experiment were successful.*
Randomization	*Describe how samples/organisms/participants were allocated into groups. If allocation was not random, describe how covariates were controlled. If this is not relevant to your study, explain why.*
Blinding	*Describe the extent of blinding used during data acquisition and analysis. If blinding was not possible, describe why OR explain why blinding was not relevant to your study.*

Did the study involve field work? ☐ Yes ☒ No

Reporting for specific materials, systems and methods

We require information from authors about some types of materials, experimental systems and methods used in many studies. Here, indicate whether each material, system or method listed is relevant to your study. If you are not sure if a list item applies to your research, read the appropriate section before selecting a response.

Materials & experimental systems

n/a	Involved in the study
☐	☒ Antibodies
☐	☒ Eukaryotic cell lines
☒	☐ Palaeontology
☐	☒ Animals and other organisms
☒	☐ Human research participants
☒	☐ Clinical data

Methods

n/a	Involved in the study
☐	☒ ChIP-seq
☒	☐ Flow cytometry
☒	☐ MRI-based neuroimaging

Antibodies

Antibodies used	The following primary antibodies were used: Rabbit anti-TET1 (Millipore, 09-872), mouse anti-TET1 (Active Motif, 91171), rat anti-TET1 (Active Motif, 61741), mouse anti-HA (Invitrogen, 26183), rabbit anti-HA (Invitrogen, 71-5500), rabbit anti-Flag (Invitrogen, PA1984B), mouse anti-Flag (sigma, F1804 and F7425), mouse anti-Tuj1 (Biolegend, 801201), rabbit anti-GFAP (Sigma, HPA056030), Rabbit anti-Egr1 (Santa Cruz Biotechnology, sc-189), mouse anti-Egr1 (Santa Cruz Biotechnology, sc-101033), rabbit normal IgG (Santa Cruz Biotechnology, sc-2027) and mouse normal IgG (Santa Cruz Biotechnology, sc-2025). The following secondary antibodies were used: goat anti-rabbit (Invitrogen, 65-6120), goat anti-mouse (Sigma, A8924).
Validation	All antibodies used were validated by the companies that provide the antibodies. Specially, in this study, TET1 antibodies were validated by the overexpression of HA-tagged Tet1s-CD in HEK293T, EGR1 antibodies were validated by using Flag-tagged EGR1-FL in HEK293T, HA and Flag antibodies were also validated by using HA-tagged Tet1s-CD and Flag-tagged EGR1-FL in HEK293T, respectively.

Eukaryotic cell lines

Policy information about cell lines

Cell line source(s)	HEK293T cells were obtained from the American Type Culture Collection (ATCC), E16.5 primary cortical neurons were isolated from E16.5 C57BL/6 mouse embryo cortices .
Authentication	E16.5 primary cortical neurons were validated by immunostaining.
Mycoplasma contamination	All cells has been tested negative for mycoplasma contamination.
Commonly misidentified lines (See ICLAC register)	No commonly misidentified cell lines were used.

Animals and other organisms

Policy information about studies involving animals; ARRIVE guidelines recommended for reporting animal research

Laboratory animals	C57BL/6 mice (male, 6 week old) and E16.5 embryos were used in this study
Wild animals	The study didn't involve wild animals
Field-collected samples	This study didn't involve samples collected from the field
Ethics oversight	All animal experiments were performed in accordance with the NIH Guide for the Care and Use of Laboratory Animals and the protocols approved by the Institutional Animal Care and Use Committee at Virginia Tech (Blacksburg, VA, USA).

Note that full information on the approval of the study protocol must also be provided in the manuscript.

ChIP-seq

Data deposition

- [x] Confirm that both raw and final processed data have been deposited in a public database such as GEO.
- [x] Confirm that you have deposited or provided access to graph files (e.g. BED files) for the called peaks.

Data access links<br>*May remain private before publication.*	To review GEO accession GSE108768:<br>https://www.ncbi.nlm.nih.gov/geo/query/acc.cgi?acc=GSE108768
Files in database submission	Fastq files for EGR1, TET1 , MACS2 peak calling narrowPeak files for , bigwig tracks for
Genome browser session<br>(e.g. UCSC)	no longer applicable

Methodology

Replicates	Biological replicates were performed with consistent results as described in the supplementary figures
Sequencing depth	ChIP libraries were sequenced in pair-end with 30-90 million 100-150 bp reads generated
Antibodies	Rabbit anti-Egr1 antibody (Santa Cruz Biotechnology, sc-189) for EGR1 ChIP-seq; mouse anti-TET1 (Active Motif, 91171), rat anti-TET1 (Active Motif, 61741, refer to as 5D6) for TET1 ChIP-seq
Peak calling parameters	Sequence reads were mapped to the mouse reference (mm10) by using Bowtie with parameters "–n 2 –l 50". EGR1 Peak calling was performed using SPP with parameters "-npeak=300000 -p=5 -savr -savp –rf", TET1 peaks were determined with MACS2 using broad parameters including the cutoff for fold change as 2 and the cutoff for q value as 1E-5
Data quality	Reads have good quality, majority read score >30
Software	FASTQC was used for quality control and assessment. Reads were trimmed with Trim_Galore (v.0.5.0) and Cutadapt(v.1.17), mapped to mouse reference genome with Bowtie2 (v2.3.4.1), peak calling uses SPP, MACS2 (v2.1.1) and Bedtools (v2.26.0)
