# Supplementary material for: EGR1 recruits TET1 to shape the brain methylome during development and upon neuronal activity
Source: Nat Commun. 2019 Aug 29;10:3892. doi: 10.1038/s41467-019-11905-3 (PMC6715719; doi:10.1038/s41467-019-11905-3)
Supplement: Supplementary file 3 — Description of Additional Supplementary Files [file 41467_2019_11905_MOESM3_ESM.docx]

**Description of Additional Supplementary Files**

File Name: Supplementary Data 1

Description: TF motif enrichment analyses for human and mouse brain co-methylated modules.

File Name: Supplementary Data 2

Description: GO enrichment analysis for genes with TSS flanking 10 kb of EGR1 peaks hypomethylated in neurons.

File Name: Supplementary Data 3

Description: Summary of TET1 ChIP-seq data.

File Name: Supplementary Data 4

Description: Statistics of RRBS and RNAseq data with annotations for differentially methylation regions and differentially expressed genes

File Name: Supplementary Data 5

Description: GO enrichment analyses for differentially expressed genes identified in Egr1KO or Tet1KO mice.

File Name: Supplementary Data 6

Description: Antibodies used for IP and western blot.

File Name: Supplementary Data 7

Description: Primer sets used in this study.

File Name: Supplementary Data 8

Description: Summary of brain “omics” data used in this study.
