# Supplementary material for: EGR1 recruits TET1 to shape the brain methylome during development and upon neuronal activity
Source: Nat Commun. 2019 Aug 29;10:3892. doi: 10.1038/s41467-019-11905-3 (PMC6715719; doi:10.1038/s41467-019-11905-3)

**Source Data: The full and original western blots used for Figure 2 and Supplementary Figure 9.** The target bands were indicated with orange arrows. The corresponding uncropped blots for Fig. 2 and Supplementary Fig. 9 were in the green boxes. “M” represents protein Marker.


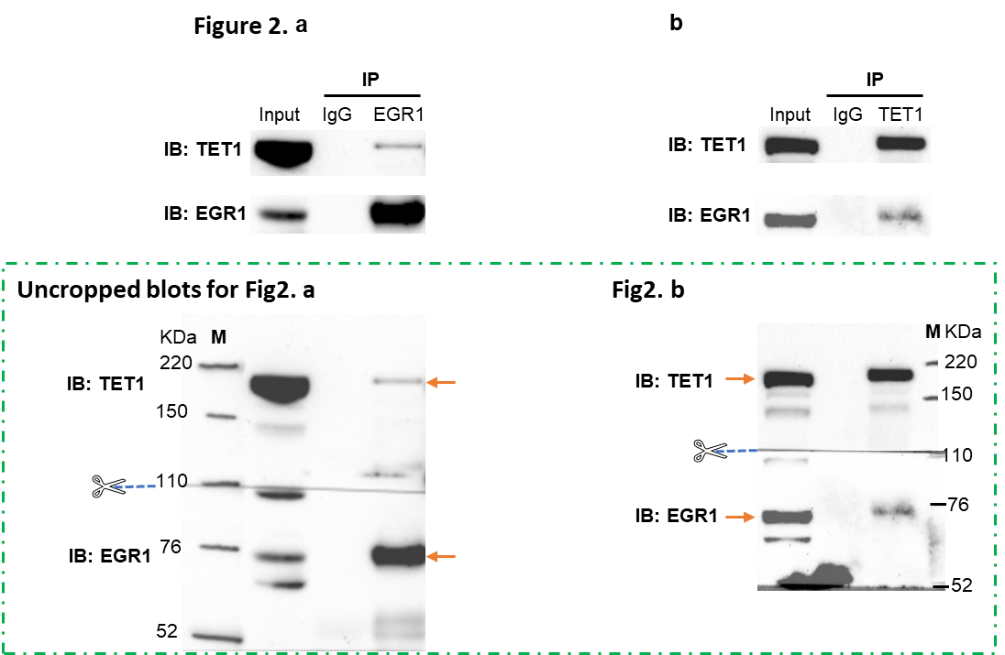


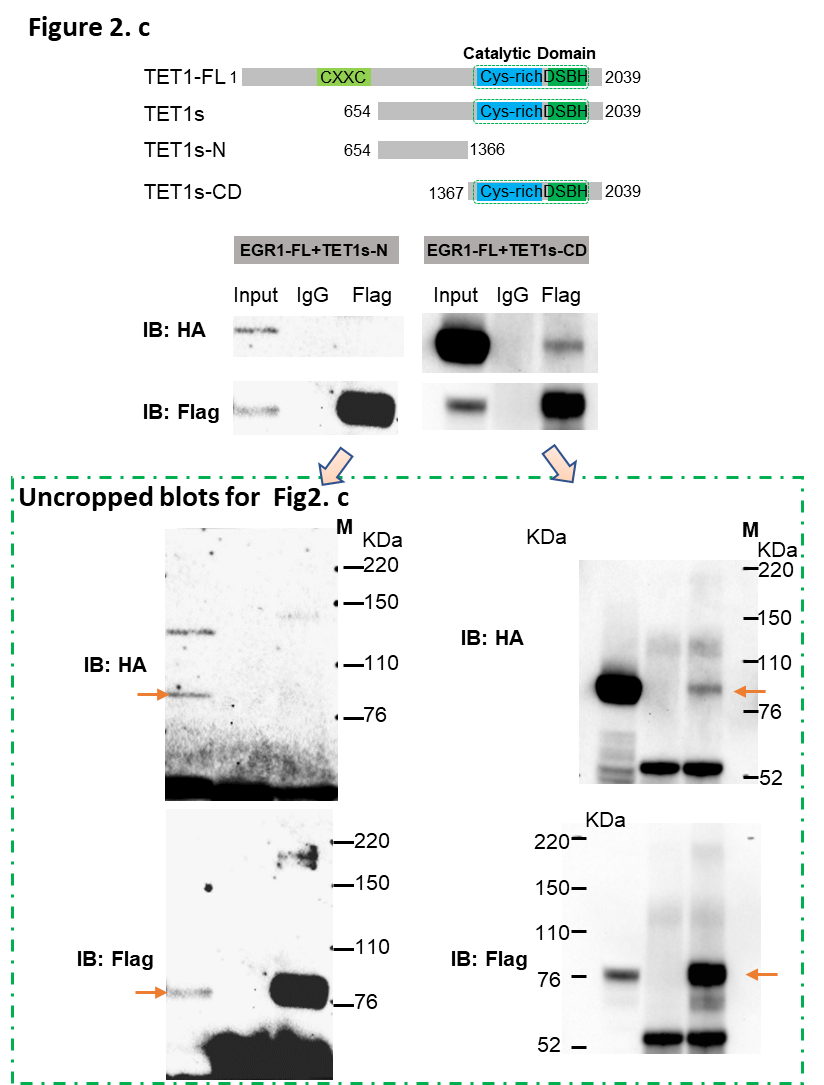


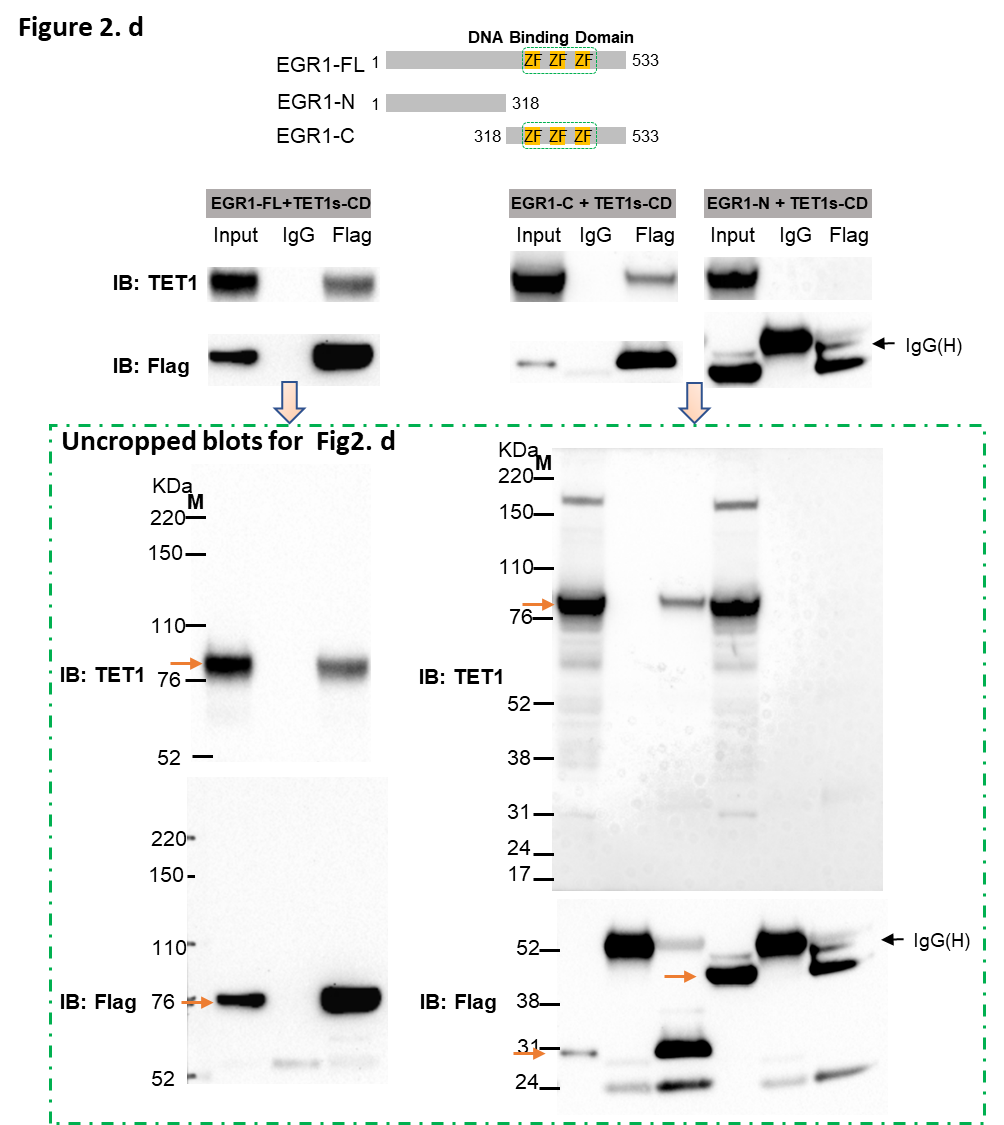


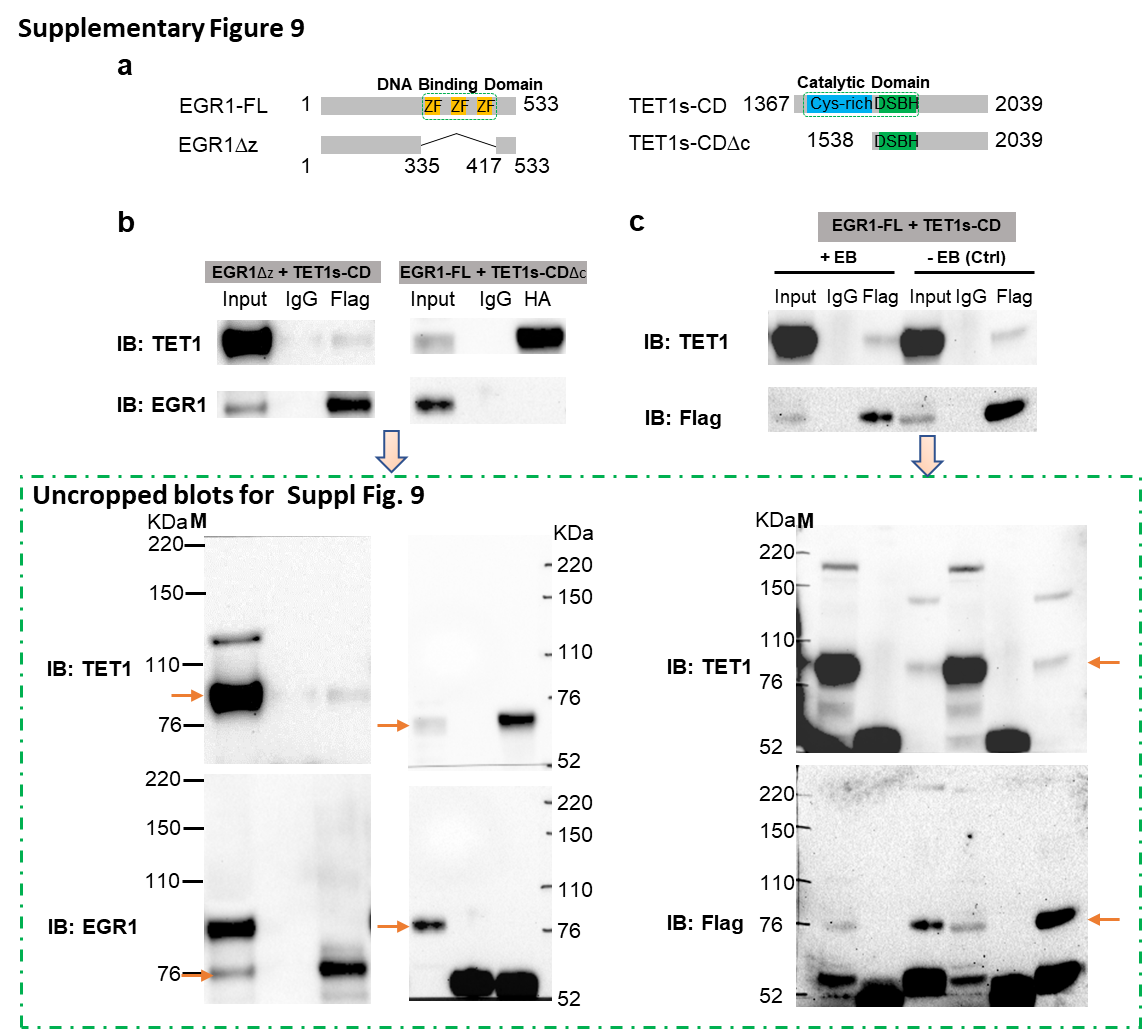

Supplement: Supplementary file 12 — Source Data [file 41467_2019_11905_MOESM12_ESM.zip › Source Data.docx]
